# Bridging Green Chemistry and Circular Economy: A Pathway to Sustainable Polyester Plastics Through Feedstock, Synthesis, and Waste Upcycling

**DOI:** 10.1002/advs.202521680

**Published:** 2026-01-26

**Authors:** Jinzhou Li, Junliang Chen, Yingbing Zhang, Li Wang, Pin Gao, Jianping Yang

**Affiliations:** ^1^ State Key Laboratory of Advanced Fiber Materials, College of Materials Science and Engineering Donghua University Shanghai 201620 China; ^2^ Department of Chemistry Shanghai Key Laboratory of Molecular Catalysis and Innovative Materials and State Key Laboratory of Porous Materials for Separation and Conversion Fudan University Shanghai 200433 China; ^3^ College of Environmental Science and Engineering Donghua University Shanghai 201620 China

**Keywords:** circular economy, green synthesis, polyester plastics, sustainable development, wastes upcycling

## Abstract

Polyester plastics have become integral to modern life due to their favorable physicochemical properties, cost‐effectiveness, and broad industrial applicability. Unfortunately, the growing consumption and disposal of polyesters have led to mounting concerns regarding the corresponding carbon emissions and persistent pollution release, presenting a critical challenge for the construction of sustainable future. Conventional mechanical recycling is constrained by polymer downgrading with limited value retention. Alternatively, emerging green catalytic technologies present promising pathways for converting polyester wastes into high‐value chemical feedstocks with reduced energy input and environmental impact. This review provides a comprehensive overview of recent advances in the sustainable synthesis and management of polyester plastics, encompassing both petroleum‐derived and bio‐based polyesters. Emphasis is placed on green monomers synthesis and polymerization strategies, catalytic valorization and upcycling approaches that boost the feedstocks recovery and enable resource circularity. Collectively, these insights aim to guide the development of a low‐carbon, high‐efficiency, and sustainable circular economy for polyester plastics.

## Graphical abstract

This review summarizes green and low‐carbon strategies throughout the entire life cycle of polyester plastics, including sustainable monomer synthesis, optimization polymerization, efficient recycling, and advanced upcycling technologies. The synergistic implementation of these strategies is expected to significantly promote the development of a circular economy for polyester plastics and effectively reduce their negative environmental impact.

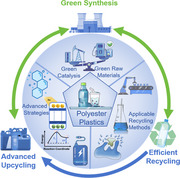

## 1 Introduction

**Figure 1:**
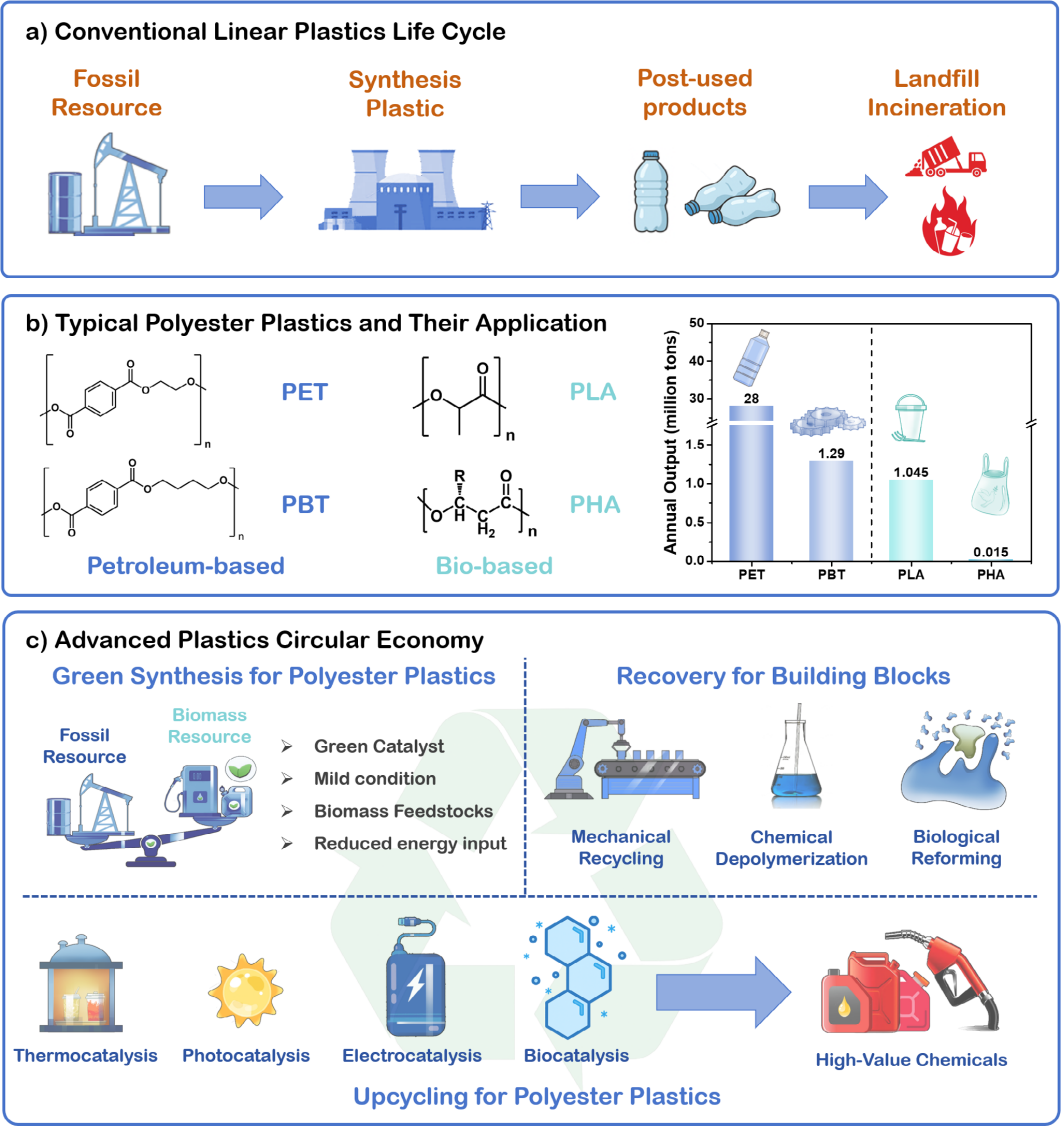
a) Conventional linear plastics life cycle. b) Application of typical polyester plastics and their output. c) Advanced technologies for polyester plastic circular economy, including: synthesis, recycling, and upcycling.

Plastics, as one of the indispensable materials in modern society, have experienced steadily increasing production driven by rapid industrialization.^[^ ^1^, ^2^, ^3^ ^]^ Although plastics bring convenience to humans’ daily lives, their huge consumption, typically short linear lifecycle, and potential toxic substances release not only exert profound impacts on global climate change but also pose risks to human health (**Figure** **1**a).^[^ ^4^, ^5^, ^6^ ^]^ Polyester‐based plastics, characterized by ester linkages in their polymer backbone, are broadly categorized into petroleum‐based and bio‐based classes according to the source of their constituent monomers (Figure 1b).^[^ ^7^, ^8^, ^9^ ^]^ Common petroleum‐based polyesters, such as polyethylene terephthalate (PET) and polybutylene terephthalate (PBT), originate from fossil resources including crude oil and natural gas.^[^ ^10^ ^]^ In contrast, the bio‐based polyesters such as polylactic acid (PLA) and polyhydroxyalkanoates (PHA) are synthesized from renewable biomass. Their monomers, notably lactic acid (LA) and hydroxy fatty acids, are typically produced via microbial fermentation or chemical conversion of plant‐derived intermediates, including corn, sugarcane, and cellulose.^[^ ^11^ ^]^ Compared to other types of plastics, polyesters often exhibit excellent physicochemical properties (mechanical strength and thermal stability), low production costs, and strong market adaptability.^[^ ^12^ ^]^ For instance, the tensile strength of PET significantly surpasses that of polyolefins like polyethylene (PE) and polypropylene (PP), while the heat distortion temperature of PBT reaches ≈200 °C—much higher than that of PE (60 °C) and polystyrene (PS, 70 °C). Such superior mechanical and thermal properties underpin the extensive industrial utilization of these polyesters.

PET is one of the most widely produced and utilized petroleum‐based polyesters, accounting for the global production of 28 million tons in 2024.^[^ ^13^ ^]^ In addition, by varying the alcohol component during synthesis, other petroleum‐based polyesters such as PBT can be derived. PET and PBT are widely used in packaging and the automotive sector, serving respectively in plastic bottles and automotive gear components. PLA, as the most widely produced bio‐based polyester, reached a global production of ≈1.045 million tons in 2024, representing 42.3% of the biodegradable plastics market.^[^ ^14^ ^]^ PLA is primarily used in sustainable products, such as disposable tableware and biodegradable plastic bags. Moreover, the ester bonds in polyesters can be chemically or biologically cleaved via hydrolysis and transesterification reactions, offering promising prospects for reclaiming building blocks for new polyesters.^[^ ^15^ ^]^ Consequently, polyesters are anticipated to become more involved in initiatives promoting circularity and sustainability in plastic management. Studies project that by 2050, polyesters plastic production could account for about 4% of global oil consumption and consume up to 3% of the global carbon budget.^[^ ^16^ ^]^ In fact, each stage during the life cycle of polyesters contributes to greenhouse gas emissions and released harmful substances, especially feedstock extraction, polymer synthesis, and waste management (disposal or recycling).^[^ ^17^ ^]^ Reducing these environmental burden thus demands innovations in life cycle management that integrates renewable feedstocks, green synthesis, and environmentally friendly end‐of‐life management, while maintains economic viability. Such sustainable direction is essential for advancing a sustainable and circular plastics economy for polyester plastics (Figure 1c). In recent years, significant progress has been made in the sustainable synthesis and upcycling of polyester plastics, particularly in the development of polyester materials derived from renewable resources and the catalytic conversion of polyester waste under mild conditions such as photo‐ or electricity‐driven processes.

In this review, we outline strategies for reducing carbon emissions across the entire life cycle of polyester plastics, with a particular focus on green monomer synthesis, polymerization strategies, catalytic valorization, and integrated upcycling approaches that enhance feedstock recovery and resource circularity. Advanced methods for high‐value recycling of polyester materials were discussed, which highlighted the design principles of catalytic systems. In addition, techno‐economic assessments (TEA) of key technological steps are illustrated. The review concludes with perspectives on the future role of polyesters in a sustainable plastics economy, offering potential solutions and research directions for addressing plastic pollution. We hope these insights can serve as a useful reference for advancing a low‐carbon and sustainable circular economy for polyester plastics.

## 2 Green Synthesis and Processing Technologies for Polyester Plastics

### 2.1 Green Synthesis Technologies for PET

**Figure 2:**
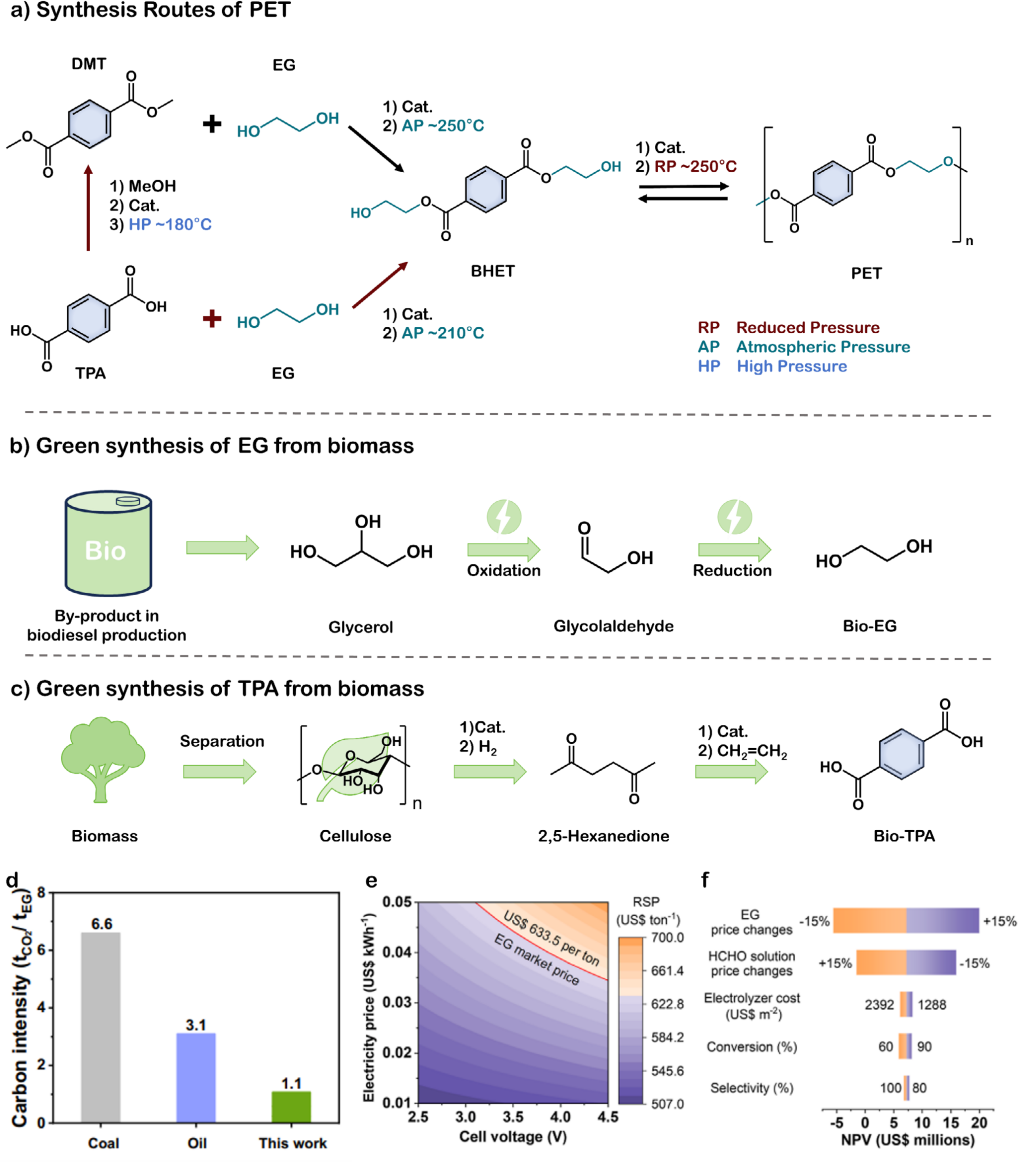
a) The synthesis methods of PET include the BHET method (Black path) and the DMT method (Red path). b) Schematic illustration of EG production from biomass glycerol. c) Schematic illustration of TPA production from biomass. d) Carbon intensity assessment for EG production by coal route, oil route, and the proposed system. Reproduced with permission.^[^ ^27^ ^]^ Copyright 2025, Springer Nature. e) TEA for producing EG. f) Sensitivity analysis of end‐of‐life NPV for producing EG. Reproduced with permission.^[^ ^28^ ^]^ Copyright 2025, American Chemical Society.

PET is synthesized via the polycondensation of two fossil‐derived monomers: TPA and EG. As shown in **Figure** **2**a, industrial PET production typically proceeds a two‐step polymerization process.^[^ ^18^ ^]^ In the first stage, TPA and EG undergo direct esterification to yield bis(2‐hydroxyethyl) terephthalate (BHET). Alternatively, TPA can first react with methanol to form dimethyl terephthalate (DMT), which subsequently undergoes transesterification with EG to generate BHET. The resulting BHET is then polycondensed into high‐molecular‐weight PET.^[^ ^19^ ^]^ Antimony‐based compounds (Sb_2_O_3_, SbAc_3_, Sb_2_(OC_2_H_4_OH)_3_) have long been served as effective catalysts in PET polycondensation, offering high activity and desirable optical properties.^[^ ^20^ ^]^ However, residual antimony in PET products has raised growing health concerns, particularly food‐grade PET. Shotyk et al. reported that the leached antimony concentrations in bottled water stored in PET containers can reach up to 1990 ng L^−1^, prompting safety concerns.^[^ ^21^ ^]^ Therefore, efforts to develop alternative catalysts that are both environmentally benign and industrially viable have been encouraged. Germanium‐based catalysts provide a low‐toxicity option but remain economically prohibitive for large‐scale applications. In contrast, titanium‐based catalysts have emerged as the most viable alternative, combining low cost, high catalytic activity, and minimal toxicity.^[^ ^22^, ^23^ ^]^ Compared with antimony systems that typically leave 250–300 ppm residues, titanium systems reduce residual content to 10–30 ppm.^[^ ^24^ ^]^ Recent advances include a titanium/magnesium composite catalyst with high activity and suppressed side reactions, and a sodium trihydroxyacetate titanate catalyst demonstrating efficient polycondensation under industrial relevant conditions.^[^ ^25^, ^26^ ^]^ The development of catalysts with minimal residue is crucial for achieving more sustainable PET production.

Beyond catalyst innovation, recent efforts have also focused on replacing fossil‐based monomers with bio‐based alternatives such as bio‐ethylene glycol (Bio‐EG) and bio‐terephthalic acid (Bio‐TPA), enabling the transition toward bio‐PET. Chi et al. proposed an electrochemical strategy for Bio‐EG synthesis from biomass‐derived glycerol.^[^ ^27^ ^]^ Glycerol is oxidized to acetaldehyde at the anode and subsequently reduced to Bio‐EG at the cathode, with potential to offset over 80 Mt of CO_2_ annually relative to conventional pathways (Figure 2b). Compared with conventional petroleum‐ and coal‐based routes, this method for producing bio‐EG results in a threefold and sixfold reduction in carbon emissions, respectively (Figure 2d). Li et al. introduced a thermoelectric system employing nanodiamond catalysts to convert formaldehyde to Bio‐EG. Studies have shown that nondefective sp^2^‐hybridized carbon serves as the primary active site for ethylene glycol (EG) production, while defective sp^2^‐hybridized carbon facilitates charge transfer. By tailoring the carbon hybridization state, the carbon‐electrolyte interface was effectively optimized, resulting in a substantial increase in EG selectivity from 37.3% to 84.1%. It achieves a FE of 92.0% at 50 °C and a current density of 1 A cm^−2^.^[^ ^28^ ^]^ Techno‐economic modeling indicates that at an applied voltage of 3.68 V, the required sale price (RSP) of Bio‐EG becomes competitive when electricity costs fall below 4.2 US¢ kWh^−1^. The conversion of formaldehyde to EG yields an estimated profit of $634 per ton of formaldehyde processed at 400 mA cm^−2^. Figure 2e shows the RSP as a function of electricity price and total cell potential at 400 mA cm^−2^. Sensitivity analysis further reveals that the net present value (NPV) is most affected by the market prices of EG and formaldehyde, indicating that the economic viability of the technology is highly susceptible to market fluctuations (Figure 2f). Dong et al. developed a Z‐scheme heterojunction photocatalyst ZnIn_2_S_4_/TiO_2_‐Cl for bio‐EG synthesis, achieving a high selectivity of 96.7% and a yield of 21.6 mmol g^−1^.^[^ ^29^ ^]^ The proposed reaction mechanism involves Cl· radicals selectively cleaving the C*─*H bond in methanol via hydrogen atom transfer (HAT) to generate ·CH_2_OH radicals, which is subsequently transformed to form EG.^[^ ^29^ ^]^ These works demonstrate the efficient production of Bio‐EG under mild reaction conditions. In addition, many companies have adopted advanced catalytic technologies for the production of bio‐based monomers. As shown in **Table** **1**, a Chinese company has initiated a pilot‐scale bio‐EG process with an annual capacity of 1000 tons.^[^ ^30^ ^]^ Sustainea (USA) has announced plans to produce and commercialize bio‐EG, with an expected capacity of 700 000 tons per year.^[^ ^31^ ^]^ These developments highlight the potential of bio‐based monomers to replace their petroleum‐derived counterparts.

**Table 1:** Announced/planned capacities for Bio‐EG, Bio‐PTA, and LA.

Chemicals	Company	Feedstock	Technical route	Annual production [ton per year]	Start year
Bio‐EG	Chinese Academy of Sciences (China)^[^ ^30^ ^]^	Corn stalks	Sugar catalytic	1000	2022
	Sustainea (USA)^[^ ^31^ ^]^	Corn	Sugar catalytic	700 000 (Project)	2024
Bio‐TPA	Mitsubishi (Japan)^[^ ^32^ ^]^	Biomass	Bio‐PX to TPA	n.r.^a)^	2023
LA/PLA	NatureWorks (USA)^[^ ^33^ ^]^	Plant sugar	Fermentation	198 000 (Project)	2025
	Emirates Biotech (UAE)^[^ ^34^ ^]^	Plant sugar	Fermentation	160 000	2025

^a)^ Abbreviations: n.r., data not reported.

In parallel, the synthesis of Bio‐TPA remains more challenging, as biomass feedstocks are predominantly aliphatic with low aromatic content and irregular distribution.^[^ ^35^ ^]^ Consequently, Bio‐TPA production typically involves multistep routes with low yields, significant by‐product formation and poor selectivity. To address these issues, Song et al. derived Bio‐TPA from lignin oil obtained from corn stalks via demethoxylation, carbonylation, and oxidation, followed by a simplified purification using solution precipitation.^[^ ^36^ ^]^ Chu et al. proposed a concise route from cellulose to 2,5‐hexanedione to p‐xylene, yielding more stable intermediates and simplifying separation compared to conventional processes (Figure 2c).^[^ ^37^ ^]^ Currently, Bio‐TPA production is limited to small‐scale demonstrations, and no exclusive large‐scale industrial plants have been reported. Commercial bio‐PET products presently contain about 30% bio‐based monomer content.^[^ ^17^ ^]^ Achieving fully bio‐based PET requires overcoming challenges in catalyst optimization, process efficiency and cost, continued advances in technology and scale‐up are expected to position Bio‐PET as a key material in the sustainable plastics market. In addition, green depolymerization of PET can also yield EG and TPA monomers, which will be discussed in detail in Section 3.

### 2.2 Green Synthesis Technologies for PLA

**Figure 3:**
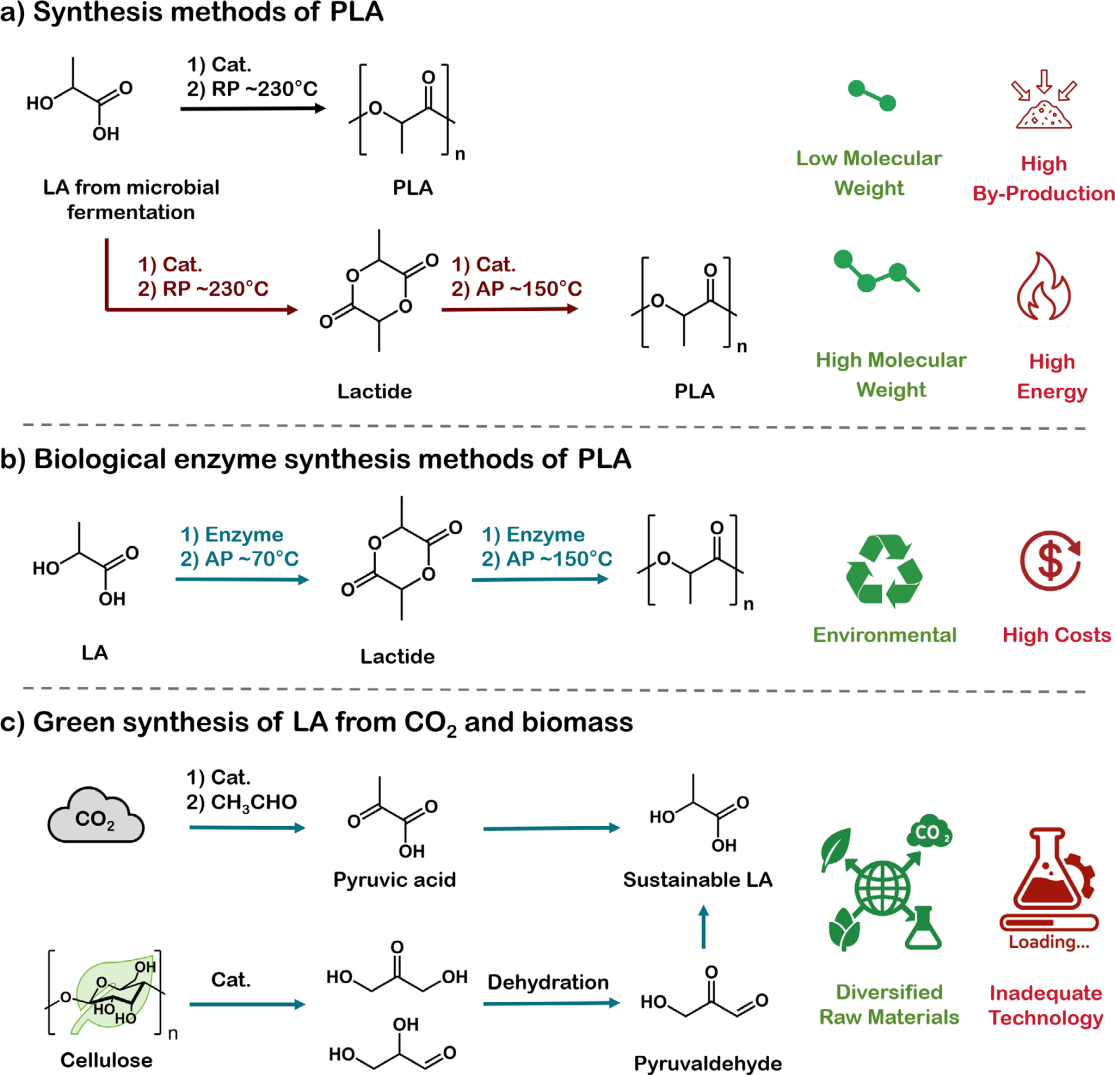
a) The synthesis methods of PLA include the direct polycondensation of LA method (Black path) and the ROP method (Red path). b) The advanced biological enzyme method for synthesis PLA. c) Life cycle environmental impacts of conventional and biomass‐based preparation of LA.

In contrast to petroleum‐based PET, which usually relies on fossil resources, PLA has found extensive use in agriculture, biomedical applications, packaging and 3D printing due to its biodegradability and favorable lifecycle metrics.^[^ ^38^, ^39^ ^]^ Given that LA, the monomer of PLA, is primarily produced via microbial fermentation of renewable biomass such as corn and sugarcane, the overall production of PLA entails lower energy inputs and a reduced environmental footprint compared to conventional petroleum‐derived polyesters. Companies such as NatureWorks and Emirates produce bio‐based LA and PLA through integrated fermentation and chemical polymerization routes, achieving an annual capacity exceeding 150 000 tons.^[^ ^33^, ^34^ ^]^ As illustrated in **Figure** **3**a, PLA can be synthesized via two main routes.^[^ ^40^ ^]^ One involves direct polycondensation of LA, a straightforward process but typically yielding low‐molecular‐weight polymers (<50 000 g mol^−1^) with considerable by‐product formation. The alternative approach proceeds through the formation of lactide, followed by ring‐opening polymerization (ROP). This method can generate high‐molecular‐weight PLA with improved control and fewer side reactions, which is favorable for industrial application.^[^ ^41^ ^]^ However, conventional ROP of LA relies on metal‐based catalysts (e.g., zinc or tin compounds), which can leave residues in the final product, raising safety concerns for biomedical and food‐related applications.^[^ ^42^, ^43^ ^]^ Additionally, the process necessitates high‐purity monomers, contributing to elevated production costs. These challenges have spurred interest in developing metal‐free catalysts, refine reaction conditions, and explore alternative polymerization strategies.

To address the limitations of metal‐catalyzed polymerization, enzymatic polymerization has emerged as a promising green alternative (Figure 3b). Operating under mild conditions, enzymatic methods provide high selectivity and eliminate metal residues, making them particularly suitable for sensitive applications. Taguchi et al. first reported a microbial one‐step synthesis of lactate‐based polyesters via PHA synthases.^[^ ^44^ ^]^ However, the produced product is a copolymer containing 6 mol% lactate and 94 mol% 3‐hydroxybutyrate (3HB), rather than a PLA homopolymer. Song et al. engineered corynebacterium glutamicum to yield PLA with 99.3 mol% lactate content, enabling an endotoxin‐free microbial production platform.^[^ ^45^ ^]^ Given lipases have shown high catalytic activity toward aliphatic polyester synthesis, Candida antarctica lipase B (CALB) has been studied for lactide ROP. The CALB active site features a catalytic triad composed of Ser105, His224, and Asp187. In this mechanism, Ser105 initiates nucleophilic attack on the carboxyl carbon of lactide to form an acyl‐enzyme intermediate, while His224 and Asp187 stabilize the transition state and facilitate proton transfer.^[^ ^46^, ^47^ ^]^ Subsequently, polymer chain growth proceeds via nucleophilic attack by hydroxyl groups on successive monomers, ultimately yielding high molecular weight of PLA. Efforts to improve polymer yield and molecular weight in enzymatic PLA synthesis have centered on optimizing reaction parameters, such as temperature, solvent environment, and enzyme immobilization strategies.^[^ ^48^, ^49^, ^50^ ^]^ Nevertheless, enzymatic ROP remains limited by sluggish kinetics, usually requiring up to 1 day to reach ≈80% monomer conversion, which is substantially longer than the metal‐catalyzed systems within few hours.^[^ ^51^, ^52^ ^]^ Overcoming these rate limitations is essential for the future development of scalable and industrially viable enzymatic routes to PLA.

Beyond polymerization, recent advances in green LA production from renewable feedstocks and even CO_2_ have opened new pathways for sustainable PLA synthesis (Figure 3c).^[^ ^53^, ^54^, ^55^, ^56^ ^]^ Ding et al. demonstrated a visible‐light‐driven ternary heterojunction photocatalyst (g‐C_3_N_4_/N‐TiO_2_/NiFe‐LDH) that modulates reactive oxygen species generation, enabling selective C*─*C bond cleavage in biomass‐derived polysaccharides and achieving LA yields of 89%–99%.^[^ ^57^ ^]^ Life cycle assessment (LCA) comparisons demonstrated that this route exhibits lower fossil resource depletion and greenhouse gas emissions than conventional fermentation processes. In addition, Albert et al. proposed an innovative carbon capture and utilization strategy for converting CO_2_ and ethanol into LA via biocatalysis.^[^ ^58^ ^]^ This process employs a cascade enzymatic system consisting of alcohol dehydrogenase, pyruvate decarboxylase, and lactate dehydrogenase, achieving 100% atom economy. This system was successfully operated under simulated flue gas conditions from a steel plant, demonstrating its substantial potential for industrial application.

Although petroleum‐based and bio‐based polyesters possess similarities in structural and performance characteristics, they diverge significantly in raw material origin, synthesis processes, and environmental performance. Petroleum‐based polyesters rely on conventional fossil resources and are currently undergoing a transition toward low‐carbon production methods through the development of catalysts with minimal residue and the incorporation of sustainable bio‐derived monomers. Bio‐based polyesters offer enhanced biodegradability and are increasingly targeted through the development of benign catalytic systems. Therefore, optimizing the feedstock renewability and polymerization efficiency of polyesters to promote their sustainable synthesis is essential to advance the sustainable design of polymer materials.

## 3 Efficient Recycling for Polyester Plastic Waste

Recycling of polyester wastes involves the systematic collection and processing of postconsumer plastics to recover their constituent monomers or yield value‐added chemicals. These products can subsequently be reintegrated into the manufacturing streams, offering the opportunity to closing the loop from production and use to recovery and regeneration. Such circularity can contribute to mitigating environmental impacts, conserving finite resources, and supporting sustainable material economies. In general, polyester recycling strategies can be broadly categorized into traditional mechanical recycling, chemical depolymerization, biological depolymerization, and advanced catalytic upcycling. In the following sections, we discuss the operational principles, mechanistic foundations and prospects for future large‐scale application of each approach. Particular attention is given to the design of effective and sustainable catalysts and reaction systems that enable selective bond cleavage to generate valuable product and circumvent the harsh condition and energy‐intensive process.

### 3.1 Mechanical Recycling

Mechanical (or physical) recycling is the widely applied method for plastic waste reprocessing. It was promoted and commercialized globally as early as the 1970s. By 2022, the number of plastics recycled through mechanical recycling had reached 54 million tons.^[^ ^59^ ^]^ This approach primarily relies on a series of physical operations that involve shredding, washing, and melting to convert plastic wastes into reusable pellets for new product manufacturing.^[^ ^60^, ^61^ ^]^ Polyesters are among the few types of plastics that are relatively well‐suited for mechanical recycling, primarily because their ester bonds remain relatively stable during the melting process. Among them, PET is especially prominent owing to its simple molecular structure, which can be efficient sorting via near‐infrared (NIR) techniques, allowing the manufacturing of recycled PET (r‐PET) with high purity.^[^ ^62^, ^63^, ^64^ ^]^ In 2022, PET achieved a global recycling rate of 22.1%. However, it should be pointed that the thermal processing in mechanical recycling can induce polymer chain scission, leading to molecular weight reduction and polymer properties degradation of r‐PET, particularly after multirepeated recycling cycles. To mitigate this problem, r‐PET is often blended with virgin PET or modified through the incorporation of functional additives, enabling the production of high‐quality films and fibers suitable for demanding applications.^[^ ^65^ ^]^ In contrast, most other polyesters tend to suffer from significant property degradation and discoloration during mechanical recycling, resulting in lower material quality.^[^ ^66^, ^67^ ^]^ For example, numerous studies have confirmed that PLA undergoes significant reductions in molecular weight and tensile strength during mechanical recycling.^[^ ^68^, ^69^ ^]^ PBT also experiences chain scission during mechanical recycling, which limits its applicability in high‐performance applications.^[^ ^70^ ^]^ Therefore, developing suitable alternative recycling technologies to replace mechanical recycling is crucial for achieving efficient recovery of polyester wastes.

### 3.2 Chemical Depolymerization

Chemical depolymerization involves the cleavage of chemical bonds such as ester linkages in polymer backbones, depolymerizing polyesters plastic wastes into yield monomers or other value‐added compounds.^[^ ^71^ ^]^ Compared with mechanical recycling, this approach enables the recovery of high‐purity monomers that can be repolymerized into virgin‐quality materials, thus serving as a cornerstone for establishing a closed‐loop recycling system. To achieve this sustainably, the development of efficient and environmentally benign depolymerization processes is of great importance. Accordingly, extensive efforts have been devoted to polyester depolymerization via hydrolysis, alcoholysis, and other emerging chemical pathways.^[^ ^72^ ^]^

#### 3.2.1 Hydrolysis

The hydrolysis of polyester plastics refers to the cleavage of ester bonds within their polymer chains in the presence of water, ultimately breaking them down into small‐molecule monomers or oligomers. Depending on the reaction conditions, hydrolysis can occur under acidic, neutral, or alkaline environments.^[^ ^73^ ^]^ Among these, neutral hydrolysis has recently gained attention as an environmentally friendly approach that avoids corrosive reagents and minimizes secondary pollution. In the case of PET, Patrícia et al. investigated the effects of various water states (saturated steam, superheated steam, saturated liquid, compressed liquid, and supercritical water) on PET hydrolysis at neutral condition and found that saturated liquid water yielded the highest depolymerization efficiency.^[^ ^74^ ^]^ Rapid heating at 5–10 °C s^−1^ enabled high TPA yields within 1 min. In contrast to 30 min required by conventional isothermal methods, this method was associated with lower environmental burdens. However, although neutral hydrolysis avoids the issue of corrosion, it is less effective in removing impurities from PET, resulting in TPA of lower purity compared to that obtained via acidic or alkaline hydrolysis.^[^ ^72^, ^75^ ^]^

**Figure 4:**
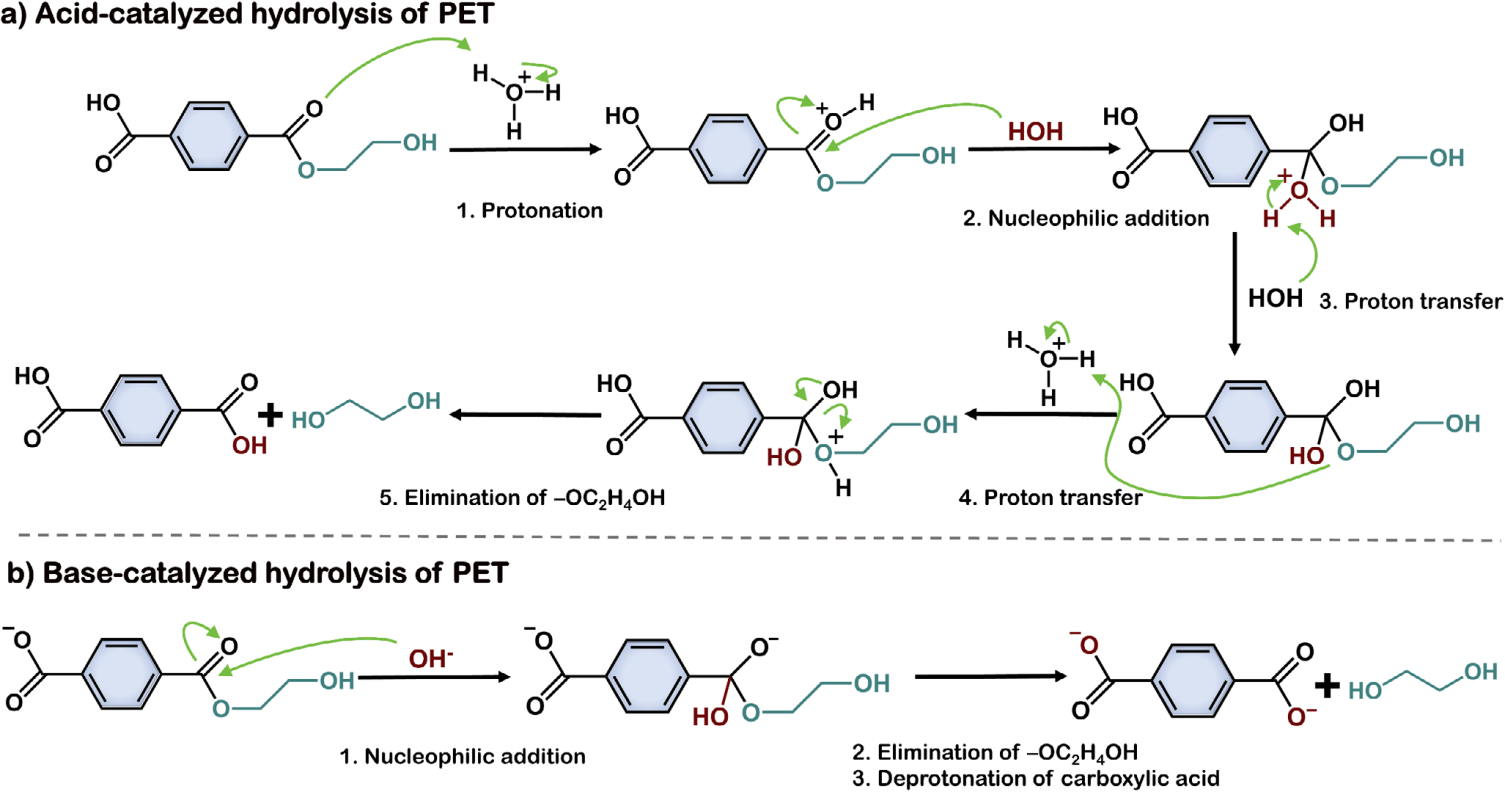
The hydrolysis mechanism of PET under acidic and alkaline conditions.

Due to the high energy consumption and relatively low product selectivity associated with neutral hydrolysis conditions, acidic and alkaline conditions are still more commonly employed for the hydrolysis of polyesters. Under acidic conditions, H^+^ attacks the ester bonds on the surface of PET, promoting depolymerization and generating corresponding monomers (**Figure** **4**a). Because TPA has limited solubility in acidic media, it can be easily separated as a solid product. Patrícia et al. compared various acids for PET hydrolysis and found that at 200 °C, organic carboxylic acids (benzoic acid, acetic acid) achieved higher TPA yields (>80%) than inorganic acids, such as nitric acid.^[^ ^76^ ^]^ In addition, aromatic carboxylic acids performed better than aliphatic acids, likely due to π–π stacking interactions between the aromatic rings of the acid and PET, which enhance bond cleavage. Under alkaline conditions, hydroxide ions attack the carbon in the ester carbonyl group, forming a tetrahedral intermediate that subsequently cleaves the ester bond to yield TPA and EG (Figure 4b).^[^ ^77^ ^]^ Sandro et al. reported that this method yields high‐purity TPA (up to 99%) at 90 °C.^[^ ^78^ ^]^

The hydrolysis mechanism of PLA is analogous to that of PET, in which the ester bonds in the polymer backbone are cleaved in the presence of water, producing soluble oligomers and LA monomers and resulting in a decrease in molecular weight. Although PLA is a biodegradable plastic, its hydrolysis proceeds slowly under ambient soil conditions, potentially causing environmental issues similar to those of conventional polyester wastes. Effective degradation of PLA generally requires elevated temperatures (>55 °C) and high humidity. Some studies have further explored the effect of pH on PLA hydrolysis: the carboxylic acid groups in the LA molecules produced during hydrolysis lower the local pH by releasing H^+^, which promotes the cleavage of ester bond and thus accelerates the reaction.^[^ ^79^ ^]^ Experimental results show that PLA can degrade rapidly under both acidic (low pH) and basic (high pH) conditions. The reaction rate is lowest at a pH around 4, which is close to the p*K*a of LA (3.84). At this pH, LA predominantly exists in its undissociated form. When the pH is above 4, LA becomes dissociated and promotes hydrolysis; when the pH is below 4, it acts as an associated acid and can further accelerate the reaction through a self‐catalytic effect.^[^ ^80^ ^]^

However, both acidic and alkaline hydrolysis suffer from issues such as high corrosiveness and environmental concerns. In addition, the requirement for high temperature and pressure, along with the need for pressure‐resistant reactors, poses challenges for industrial‐scale implementation due to high energy consumption and equipment costs. Further research is needed to optimize reaction conditions, develop efficient catalytic systems, and innovate reactor design to enhance the feasibility of hydrolysis in practical applications.

#### 3.2.2 Alcoholysis

The alcoholysis process of polyester wastes primarily involves the cleavage of ester bonds using alcohols (methanol and ethanol) in the presence of catalysts, generating monomers.^[^ ^84^ ^]^ As one of the most reliable chemical recycling strategies, alcoholysis plays a vital role in the depolymerization and recovery of polyester plastic wastes. PET manufacturers such as Hoechst, DuPont, and Eastman–Kodak have adopted alcoholysis processes for the recycling of waste PET, generating DMT and EG.^[^ ^85^ ^]^ In general, alcoholysis conducted without catalysts demands harsh reaction conditions, including elevated temperatures and pressures, which in turn result in increased costs and reduced product selectivity.^[^ ^86^, ^87^ ^]^ Therefore, the development of efficient catalysts to alleviate harsh reaction conditions and improve product selectivity is crucial for the development of alcoholysis.^[^ ^8^ ^]^ **Table** **2** summarizes various catalysts employed for the alcoholysis of PET. It can be observed that the addition of catalysts significantly reduces the required reaction temperature and pressure, while simultaneously enhancing the yield of DMT. Besides PET, PLA can also be depolymerized via alcoholysis. In this process, the hydroxyl groups of alcohols act as nucleophiles, attacking the carbonyl carbon sites in the PLA backbone and facilitating transesterification reactions. Alcohols with stronger nucleophilicity, such as methanol or ethanol, exhibit better depolymerization efficiency toward PLA through alcoholysis.^[^ ^88^ ^]^ Jack M. et al. developed zinc and magnesium complexes based on a tridentate ONN ligand. These catalysts enabled the conversion of PLA to methyl lactate at 50 °C within 30 min, achieving a yield of 85%. This represents one of the fastest catalytic systems reported to date for PLA alcoholysis.^[^ ^89^ ^]^

**Table 2:** Catalysts are used for the alcoholysis and glycolysis of PET.

	Catalyst	Solv.^a)^	Temp. [°C]	Press. [MPa]	Time [min]	PET Conv. [%]	Prod. [%]
Alcoholysis	None^[^ ^87^ ^]^	MA	220	2.0	50	100	79.1
	PCNS^[^ ^95^ ^]^	MA	185	0.1	360	n.r.	85
	Cu/SiO_2_^[^ ^96^ ^]^	MA	200	0.1	90	92.4	99
	Mg/NaY^[^ ^97^ ^]^	MA	200	0.1	30	99	91
	ChCl/ Zn(OAc)_2_ DES^[^ ^81^ ^]^	MA:CN = 1:1	120	0.1	120	100	80
	NEt_3_^[^ ^98^ ^]^	MA:PhMe = 1:1	200	0.1	180	100	88
Glycolysis	Urea/ZnCl_2_^[^ ^99^ ^]^	EG	170	0.1	30	100	83
	ZIF‐8^[^ ^100^ ^]^	EG	180	0.1	240	91.7	76.1
	ZnO^[^ ^82^ ^]^	EG	196	0.1	40	100	97.3
	FeCl_3_‐CoFe_2_O_4_^[^ ^101^ ^]^	EG	200	0.7	60	100	95.4

^a)^ Abbreviations: Solv., Solvent; Temp., Temperature; Press., Pressure; PET Conv., PET Conversion; Prod., Production.

**Figure 5:**
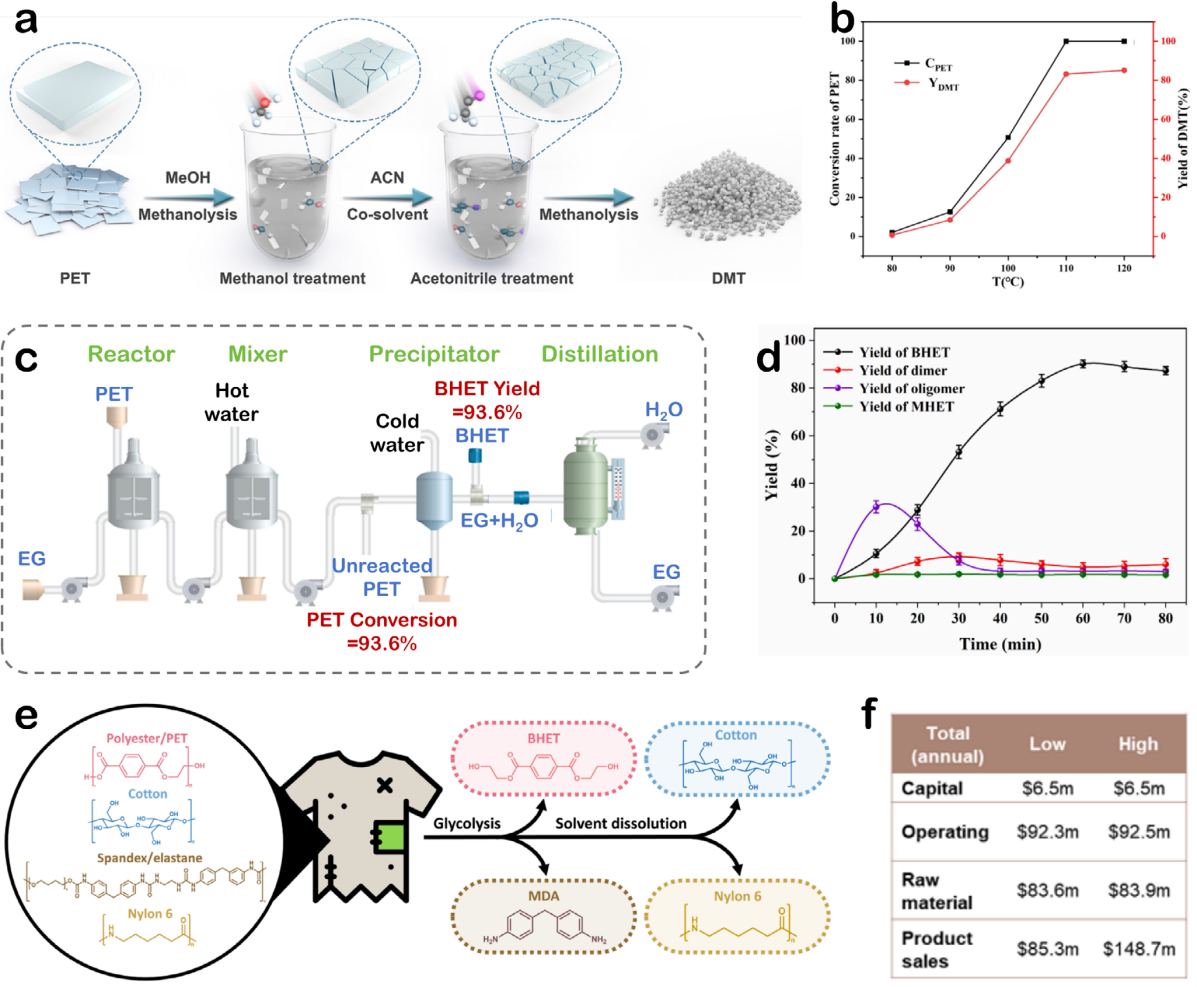
a) Schematic illustration of the alcoholysis of PET by cosolvent strategy. b) Effect of different reaction temperatures on the methanolysis of PET. Reproduced with permission.^[^ ^81^ ^]^ Copyright 2023, American Chemical Society. c) The flowsheet for PET glycolysis. d) The variations in BHET, MHET, and dimer content on PET glycolysis. Reproduced with permission.^[^ ^82^ ^]^ Copyright 2024, Elsevier. e) Real mixed plastic wastes (polyester, cotton, spandex, and nylon) using microwave assisted glycolysis and solvent dissolution. f) TEA analysis including capital, operating, raw material costs, and product sales. Reproduced with permission.^[^ ^83^ ^]^ Copyright 2024, AAAS.

In addition, the use of cosolvent strategies can further reduce the reaction temperature and improve the conversion efficiency. Tang et al. proposed an acetonitrile‐assisted cosolvent approach for PET depolymerization (**Figure** **5**a).^[^ ^81^ ^]^ When combined with a deep eutectic solvent catalyst composed of choline chloride and zinc acetate, this method enabled complete depolymerization of PET at 120 °C within 2 h. A conversion rate of 100% was achieved under these conditions (Figure 5b). In the alcoholysis of PLA, a cosolvent strategy can also be employed to enhance conversion rates and product selectivity. Due to the poor solubility of PLA in alcohols, additional organic solvents are often introduced to improve PLA dissolution and promote sufficient contact between PLA and alcohol, thereby accelerating the reaction rate.^[^ ^90^ ^]^ Commonly used cosolvents include tetrahydrofuran (THF), dichloromethane (DCM) and acetone.^[^ ^91^ ^]^

Glycolysis, a specific form of alcoholysis, utilizes polyalcohols (EG, propylene glycol, and 1,4‐butanediol (BDO)) as depolymerizing solvents. Through transesterification, PET is broken down into monomers or oligomers, such as BHET. Compared to traditional alcoholysis, glycolysis offers lower reaction temperatures and higher conversion efficiencies for PET, making it a more energy‐efficient and effective depolymerization strategy for PET (Table 2). Furthermore, the BHET product can be directly reused for PET resynthesis or in the production of modified polyesters, simplifying the overall recycling process. Ao et al. reported a one‐step low‐temperature method for synthesizing ZnO nanoparticles, which were applied to efficiently catalyze the glycolysis of PET for chemical recycling.^[^ ^82^ ^]^ The scalability of the process was further validated in a pilot‐scale experiment, yielding a PET conversion of 97.4% and a BHET yield of 93.1% (Figure 5c). Figure 5d shows that the highest BHET yield was obtained at 60 min, after which the extended reaction time led to reversed polymerization of BHET into dimers, thereby decreasing the yield of the target product. Besides PET, another petroleum‐based polyester PBT can also be chemically recycled via glycolysis.^[^ ^92^, ^93^ ^]^ Peng et al. dispersed ZnO nanoparticles into BDO to form a stable nanodispersion catalyst.^[^ ^94^ ^]^ Under mild conditions (200 °C, atmospheric pressure, 45 min) complete conversion of PBT was achieved, yielding bis(4‐hydroxybutyl) terephthalate (BHBT) with up to 98% yield.

Nevertheless, real‐world polyester wastes often exist as mixed materials, which significantly complicates their chemical recycling. Developing effective chemical recycling strategies for the separation of mixed plastic waste is of great importance.^[^ ^102^, ^103^ ^]^ To address this challenge, Erha et al. proposed an innovative chemical recycling method for mixed textile waste.^[^ ^83^ ^]^ The approach employs microwave‐assisted catalytic glycolysis as the core step under the catalysis of ZnO at 210 °C (Figure 5e), selective conversion of the mixed components is achieved. PET is efficiently depolymerized into its monomer BHET, while spandex is degraded into diphenylmethane‐containing compounds and polyols. Subsequently, a solvent separation step is used to process the remaining solid residues. Treatment with 90% formic acid (FA) at room temperature dissolves nylon, while cotton remains insoluble. The dissolved nylon can be recovered by distillation, and the undissolved cotton is separated via filtration. This study is the first to report a comprehensive chemical recycling route for a four‐component plastic mixture. TEA further demonstrated its potential feasibility, offering an innovative solution for the chemical recycling of complex mixed plastic wastes (Figure 5f). Overall, both alcoholysis and glycolysis offer viable routes for establishing a circular PET economy. While alcoholysis broadens product applications and enables value‐added utilization, glycolysis is suitable for monomer recovery to support closed‐loop production, collectively advancing plastic sustainability.

**Figure 6:**
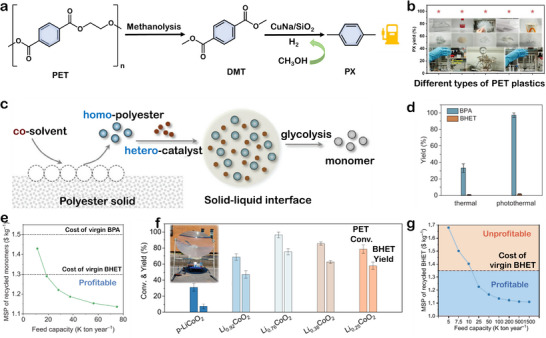
a) Strategy for the methanolysis and coupling of PET wastes. b) Different types of PET plastics are used in combination with methanolysis and selective hydrogenolysis to produce PX. Reproduced with permission.^[^ ^104^ ^]^ Copyright 2022, Springer Nature. c) Schematics solvent‐assisted heterogenous catalysts‐driven polyester glycolysis routes. d) Catalytic performance comparison of GB‐rich CeO_2_ catalysts at 140 °C under thermal and photothermal conditions. e) Effect of plant scale on the MSP of recycled monomers. Reproduced with permission.^[^ ^105^ ^]^ Copyright 2025, Wiley. f) Conversion of PET and yield of BHET over Li_1‐_ *_x_* CoO_2_ insert: portable outdoor test system. g) BHET MSP as a function of process variables of MTPY. Reproduced with permission.^[^ ^106^ ^]^ Copyright 2024, Springer Nature.

Coupling alcoholysis and glycolysis with other reactions enables further product diversification. For example, Gao et al. developed a Cu/SiO_2_‐catalyzed process that converts PET into para‐xylene (PX) and EG using methanol as both solvent and hydrogen donor, thereby eliminating the need for external hydrogen (**Figure** **6**a).^[^ ^104^ ^]^ This process integrates alcoholysis with selective hydrogenolysis to produce PX and EG. To demonstrate practical feasibility, the authors selected several common types of PET plastic wastes found in beach sediments from Phuket, Thailand (Figure 6b), and applied this process to produce 181 kg of PX and 105 kg of EG per ton of PET waste. Similarly, Li et al. proposed a synergistic catalytic system coupling PET alcoholysis with CO_2_ hydrogenation.^[^ ^107^ ^]^ In this process, the methanol produced from CO_2_ hydrogenation was directly consumed during PET alcoholysis, driving the reaction forward and enabled higher methanol yields than thermodynamically expected. Additionally, the alcoholysis product DMT was further hydrogenated into dimethyl 1,4‐cyclohexanedicarboxylate, shifting the reaction equilibrium forward and achieving an EG yield of 88.6%. This coupled approach exemplifies how alcoholysis can be integrated into multistep catalytic systems to realize both carbon circularity and energy efficiency. Despite the promising potential of alcoholysis for closed‐loop recycling, challenges such as high energy demands, the need for elevated temperatures and pressures and complex downstream separation processes necessitate further technological advancements.

Recently, advanced photothermal catalytic strategies have emerged as promising alternatives for driving the alcoholysis and glycolysis of polyester plastics.^[^ ^108^ ^]^ In photothermal catalysis, photothermal catalysts harvest solar energy to increase local temperatures via light absorption, thereby reducing reliance on fossil‐fuel‐derived heat. This localized heating concentrates thermal energy at the catalytic sites, enhancing energy utilization efficiency. Liu et al. developed a heterogeneous photothermal catalyst based on grain boundary‐rich cerium oxide (GB‐rich CeO_2_) nanoparticles.^[^ ^105^ ^]^ The introduction of cosolvents effectively addressed interfacial mass transfer limitations, enhancing plastic solubility and overall depolymerization performance (Figure 6c). By engineering the grain boundaries to regulate oxygen vacancies, authors achieved selective and stepwise recycling of mixed plastics composed of polycarbonate (PC) and PET. Compared with conventional thermocatalysis, the BPA yield during PC depolymerization at 140 °C was 2.9 times higher (Figure 6d), which was attributed to the specific adsorption and activation of carbonyl groups in PC by oxygen vacancies, as well as the locally enhanced reaction kinetics driven by the photothermal effect. Economic analysis (Figure 6e) indicated that the cost‐effectiveness of photothermal recycling is strongly dependent on production scale. This approach demonstrates notable cost advantages at large scale and holds great potential as a key solution for advancing a circular plastic economy. In addition, Lou et al. repurposed spent lithium cobalt oxide (LiCoO_2_) cathode materials from waste lithium‐ion batteries as photothermal catalysts for the depolymerization of polyester plastic wastes.^[^ ^106^ ^]^ The optimized catalyst Li_0.76_CoO_2_ achieved a BHET yield 10.24 times higher than that of pristine LiCoO_2_ (Figure 6f). In outdoor experiments (Figure 6f inset), a Fresnel lens was used to concentrate sunlight, heating EG to 197 °C within 50 s and enabling complete PET conversion in 20 min. As shown in Figure 6g, the minimum selling price of BHET was estimated to be 1.135$ kg^−1^, which is lower than the BHET market price range of 1.60$ kg^−1^, suggesting that further cost reductions are expected upon scale‐up. Future research should focus on designing efficient photothermal catalysts and optimizing catalytic mechanisms to enhance selectivity and energy efficiency, while exploring the integration of alcoholysis and glycolysis with other reactions to diversify high‐value products and promote a sustainable circular economy for polyester plastics.

#### 3.2.3 Aminolysis

Aminolysis refers to the cleavage of polymer chains in the presence of ammonia or amine compounds, yielding small molecules containing amide or amino groups. Owing to the irritant, toxic and corrosive nature of these reagents, aminolysis requires high standards of equipment sealing and corrosion resistance, which has limited its broader application.^[^ ^109^ ^]^ Although hydrolysis and alcoholysis are more commonly employed for PET depolymerization, aminolysis can also produce valuable products, such as bis(2‐hydroxyethyl) terephthalamide (BHETA). Ji et al. utilized trifluoroacetic acid (TFA) as a preactivator, where synergistic F*─*H bonding disrupted the crystalline structure and intermolecular interactions of PET. Under ambient or mild conditions, PET was aminolyzed to yield nine different terephthalamide derivatives.^[^ ^110^ ^]^ This method was also applicable to polyester blends and successfully achieved kilogram‐scale depolymerization of real PET waste, offering a new strategy for the circular plastic economy, particularly for complex or multicomponent waste streams. Poderyte et al. demonstrated catalyst‐free aminolysis of PET with ethylenediamine, producing bis‐aminoamide (BAETA), which served as a high‐performance solid CO_2_ adsorbent (3.4 mol kg^−1^).^[^ ^111^ ^]^ This approach not only directly valorizes PET waste into functional high‐value materials but also provides a “two‐in‐one” solution to both plastic pollution and greenhouse gas emissions.

In addition to PET, the aminolysis of PLA has also attracted attention. Jetawat et al. employed a microwave‐assisted aminolysis reaction to depolymerize postconsumer PLA, yielding a series of LA oligomers containing amide linkages. In this process, ethylenediamine was used as the nucleophilic reagent, and the aminolysis of PLA was carried out in a microwave reactor at 180 °C for 10 min under 200 psi. The sharp decrease in the average degree of polymerization confirmed that the long PLA chains were successfully cleaved. The obtained products, especially the LA oligomers bearing amino and hydroxyl functionalities, can serve as versatile precursors for the synthesis of high‐value bio‐based materials, such as polyurethanes, offering a promising route for the valorization of PLA waste. Aminolysis thus provides not only an efficient chemical route for polyester depolymerization but also generates reactive intermediates endowed with amino and hydroxyl groups, which are highly desirable for subsequent chemical transformations. Compared with hydrolysis or alcoholysis, aminolysis end products possess greater functional diversity and reactivity, enabling their direct utilization in the synthesis of polyurethanes, polyamides, or epoxy curing agents. This dual advantage—simultaneous degradation and functionalization—positions aminolysis as a bridge between chemical recycling and upcycling. With the development of greener processes, such as microwave‐assisted systems, ionic liquids, or heterogeneous catalysts, aminolysis is expected to play an increasingly important role in advancing closed‐loop and value‐added recycling of polyester wastes.

### 3.3 Biological Treatment

**Figure 7:**
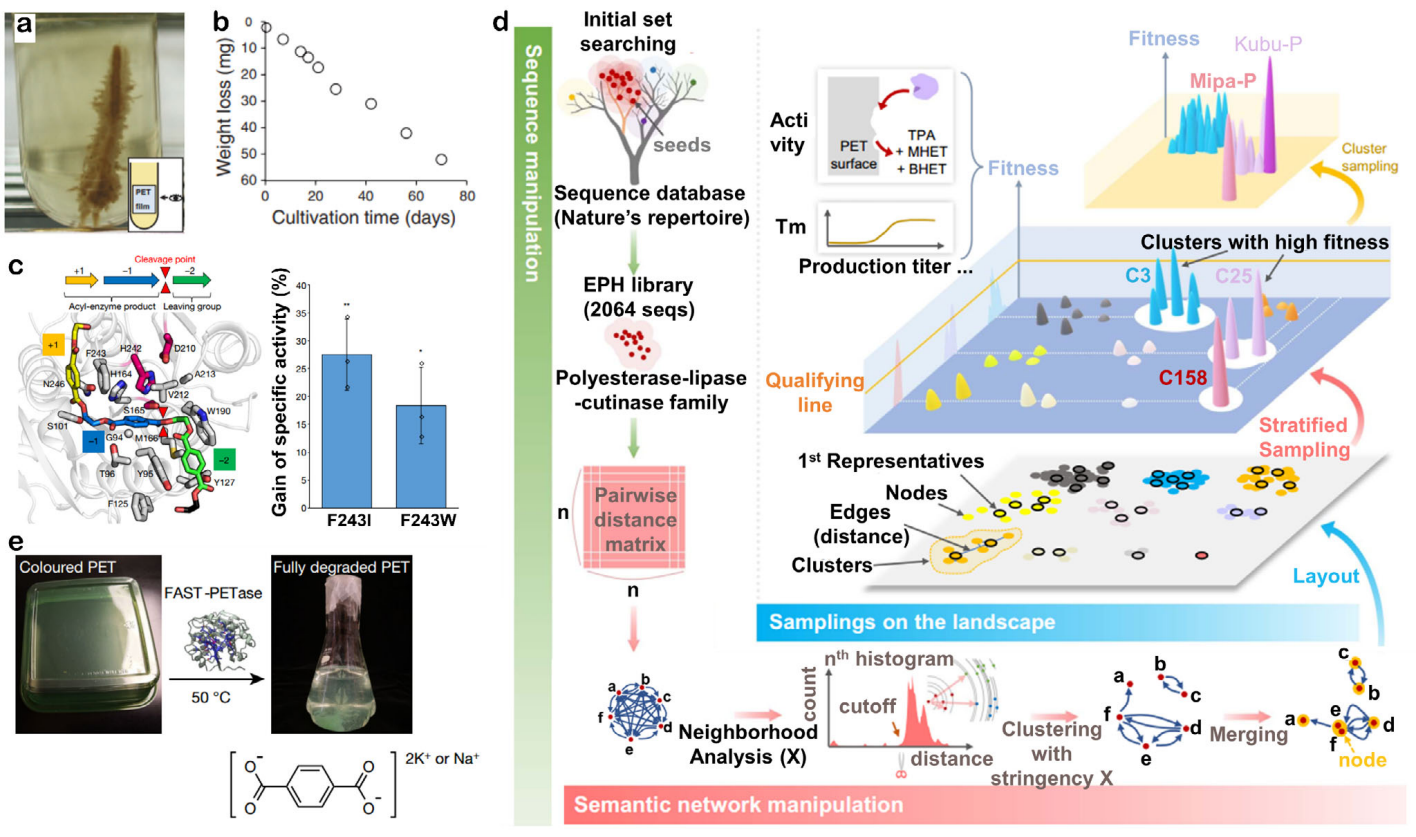
a) Digital photograph of microbial growth on PET. b) Time course of PET film degradation. Reproduced with permission.^[^ ^112^ ^]^ Copyright 2016, AAAS. c) Structural model of 2‐HE(MHET)_3_ (colored stick model) docked in wild‐type LCC (gray ribbon). Calculated percentage improvement in specific activity of Pf‐PET depolymerization by the F243I and F243W variants compared with wild‐type LCC at 65 °C. Reproduced with permission.^[^ ^113^ ^]^ Copyright 2020, Springer Nature. d) Using FAST‐PET enzymes to depolymerize post‐consumer colored plastic waste. Reproduced with permission.^[^ ^114^ ^]^ Copyright 2022, Springer Nature. e) Landscape and profiling procedures. Reproduced with permission.^[^ ^115^ ^]^ Copyright 2025, AAAS.

Enzymatic biodegradation has attracted growing interest because it operates under mild conditions and generates minimal environmental impact. Compared with polyolefins, polyesters are more susceptible to enzymatic attack owing to their ester linkages. Several natural enzymes (proteases and lipases) have been identified for polyester degradation.^[^ ^116^ ^]^ These enzymes are capable of recognizing and cleaving specific chemical bonds within plastic polymer chains, gradually breaking them down into smaller and soluble molecules. Shosuke et al. were the first to report that *Ideonella sakaiensis* secretes two enzymes, PETase and MHETase, which act synergistically to facilitate the degradation of PET.^[^ ^112^ ^]^ This strain can utilize PET plastic as its primary carbon and energy source, forming a biofilm on the PET surface (**Figure** **7**a). The two natural enzymes secreted by *Ideonella sakaiensis* can degrade PET films at a rate of 0.13 mg cm^−2^ day^−1^ under 30 °C (Figure 7b). However, natural enzymes generally exhibit poor thermal stability and low activity, limiting their industrial application. To overcome these shortcomings, significant progress has been made in enzyme engineering. Tournier et al. systematically tested a panel of PETases and identified LCC (Leaf Compost Cutinase) as the most active enzyme at 65 °C.^[^ ^113^ ^]^ By applying molecular docking and site‐saturation mutagenesis, they engineered LCC to enhance both catalytic activity and thermal stability. The best variants, ICCG and WCCG achieved over 90% PET depolymerization within 10 h at 72 °C (3 mg enzyme g^−1^ PET), with a productivity of 16.7 g L^−1^ h^−1^—substantially higher than previously reported enzymes (Figure 7c). Notably, feasibility was demonstrated in a 150 L pilot‐scale reactor, where the enzyme cost accounted for only ≈4% of the overall recycling cost (25 USD kg^−1^ enzyme), underscoring the industrial potential of enzyme engineering for plastic recycling and circular economy. In another work, Seo et al. constructed a large “esterase–lipase–cutinase” family library comprising over 2000 sequences.^[^ ^115^ ^]^ Through hierarchical sampling, they validated that more than half of the tested enzymes exhibited detectable PET degradation activity, importantly, discovered three previously unexplored high‐potential clusters (C3, C25, C158). From these, two high‐performance enzymes, Mipa‐P and Kubu‐P, were characterized, and further rational cross‐template engineering yielded an ultrastable mutant, Kubu‐PM12 (*T*_m_ > 99.9 °C), which exhibited excellent degradation efficiency and durability under harsh industrial conditions (Figure 7d). This study greatly expands the enzyme repertoire for bioplastic recycling and offers a powerful framework for future targeted mining of robust enzymes.

Recently, machine learning (ML) has emerged as a transformative strategy for accelerating enzyme engineering. Unlike conventional protein engineering, which relies on random mutagenesis or limited rational design followed by extensive experimental screening, ML‐based approaches can rapidly identify beneficial mutations. For example, Lu et al. applied the structure‐guided deep learning model MutCompute to redesign PETase.^[^ ^114^ ^]^ By predicting mismatched amino acid sites in the local structural environment, they introduced multiple mutations to create FAST‐PETase, which exhibited superior hydrolytic activity across a wide temperature (30–50 °C) and pH (neutral to mildly alkaline) range. This variant enabled near‐complete degradation of untreated postconsumer PET products, including food, beverage, and pharmaceutical packaging (Figure 7e). This study underscores the powerful synergy between machine learning and biocatalyst design in achieving efficient and scalable enzymatic recycling.

Although PLA is classified as a biodegradable plastic, its degradation in natural environments is extremely slow. Incomplete degradation may lead to the accumulation of PLA microplastics, while complete mineralization ultimately produces CO_2_ and H_2_O, potentially contributing to environmental burdens, such as microplastic pollution and greenhouse gas emissions. Therefore, developing efficient enzymes for depolymerizing PLA into LA is of significant interest. To address this, Williams et al. first reported a *proteinase K* that derived from the fungus Triticum aestivum to degrade PLA fibrous granules in 1981.^[^ ^117^ ^]^ MacDonald et al. further investigated the depolymerization mechanism of PLA by *proteinase K*. Due to the structural similarity between L‐lactide units in PLA and the side chains of natural amino acids (L‐alanine), leading proteases to mistake ester (C*─*O) bonds for peptide (C*─*N) bonds.^[^ ^118^ ^]^ Consequently, *proteinase K* has been regarded as one of the most effective enzymes for PLA depolymerization. The degradability of PLA, however, strongly depends on its stereochemistry and molecular weight—L‐type PLA is more susceptible to enzymatic attack, whereas D‐type PLA is less accessible to enzymes.^[^ ^119^ ^]^

Progress has been made in the enzymatic degradation of low‐molecular‐weight PLA, but the depolymerization of high‐molecular‐weight PLA remains challenging due to severe chain entanglement and limited accessibility of chain ends to enzymes.^[^ ^118^ ^]^ To address this, Li et al. isolated an effective PLA‐degrading bacterial strain, *Amycolatopsis orientalis ssp. orientalis*, from soil and successfully identified three enzymes: PLAase I, PLAase II, and PLAase III.^[^ ^120^ ^]^ Among them, PLAase III exhibited a PLA hydrolytic activity of 100%, which is significantly higher than that of *proteinase K*. These PLAases displayed stronger affinity toward PLA substrates and significantly lower activity toward protein substrates, indicating a higher substrate specificity for polyester‐type materials. Moreover, all three PLAases demonstrate remarkable degradation capabilities against high‐molecular‐weight, semicrystalline PLA films, suggesting that they may possess more hydrophobic binding sites or domains that enable effective attachment to PLA surfaces, facilitating efficient hydrolysis.

Recycling technologies for polyester wastes are evolving toward greater efficiency and sustainability. Mechanical recycling is the most widely adopted method as it offers simplicity and low energy consumption. However, its major limitation lies in the progressive deterioration of material properties with each recycling cycle. Chemical recycling methods can depolymerize polyesters into their monomers or value‐added chemicals, offering the potential for closed‐loop recycling. Nevertheless, these processes often require harsh reaction conditions, high energy input and involve complex product separation, posing challenges for industrial scalability. Enzymatic recycling operates under mild conditions and is environmentally friendly, making it suitable for applications with strict safety requirements. However, current enzymatic systems face limitations in activity and stability, along with high costs and slow degradation rates, restricting their applicability to large‐scale processing. Future research addresses these challenges by advancing catalyst and enzyme design, optimizing reaction systems and designing more efficient processes. These efforts are essential to enable the polyester recycling technologies toward truly sustainable and scalable industrial applications.

## 4 Advanced Upcycling Strategies for Polyester Plastic Waste

Advanced upcycling of post‐used polyesters has garnered increased attention as an effective strategy for plastic waste valorization. In contrast to traditional closed‐loop recycling, upcycling approaches aim to convert polyester wastes into fuels or high‐value chemicals through catalytic processes, thereby providing an alternative pathway that circumvents challenges, such as complex monomer separation and deterioration of mechanical properties.^[^ ^121^, ^122^ ^]^ Currently, thermal catalysis, photocatalysis, electrocatalysis and biocatalysis represent the major routes in plastic upcycling and have witnessed rapid advancement.^[^ ^123^, ^124^ ^]^

Thermal catalysis, the most industrially established protocol, employs high temperatures and pressures under an inert or reducing gas atmosphere to trigger the scission of polymers’ chemical bonds. Its compatibility with diverse polymer types, including polyesters and polyolefins, along with its relatively fast reaction kinetics, has facilitated early commercialization.^[^ ^125^ ^]^ However, the high energy input and associated carbon emissions present critical challenges to its sustainability. Photocatalysis leverages semiconductor‐based catalysts to generate electron–hole pairs under light irradiation, initiating the oxidative cleavage of polymer chains under ambient conditions. Electrocatalysis leverages renewable electricity to drive redox reactions at electrode surfaces. Both photocatalysis and electrocatalysis offer precise control over reaction pathways while minimizing energy input and environmental impact, positioning them as inherently low‐carbon and potentially decentralized strategies for waste polyesters valorization. Biocatalysis offers a sustainable route for plastic upcycling, where engineered microorganisms convert depolymerized monomers into high‐value products through metabolic processes in bioreactors. Their compatibility with ambient operating conditions, coupled with high tunability and intrinsic operational safety, has made these catalytic strategies increasingly attractive for the development of sustainable polyesters upcycling technologies. In this Section 4, we emphasized the design of catalysts and reaction systems to enable the efficient upcycling of PET and PLA, thereby providing strong support for a more sustainable polyester economy.

### 4.1 Thermocatalytic Reforming of Polyester Wastes

**Figure 8:**
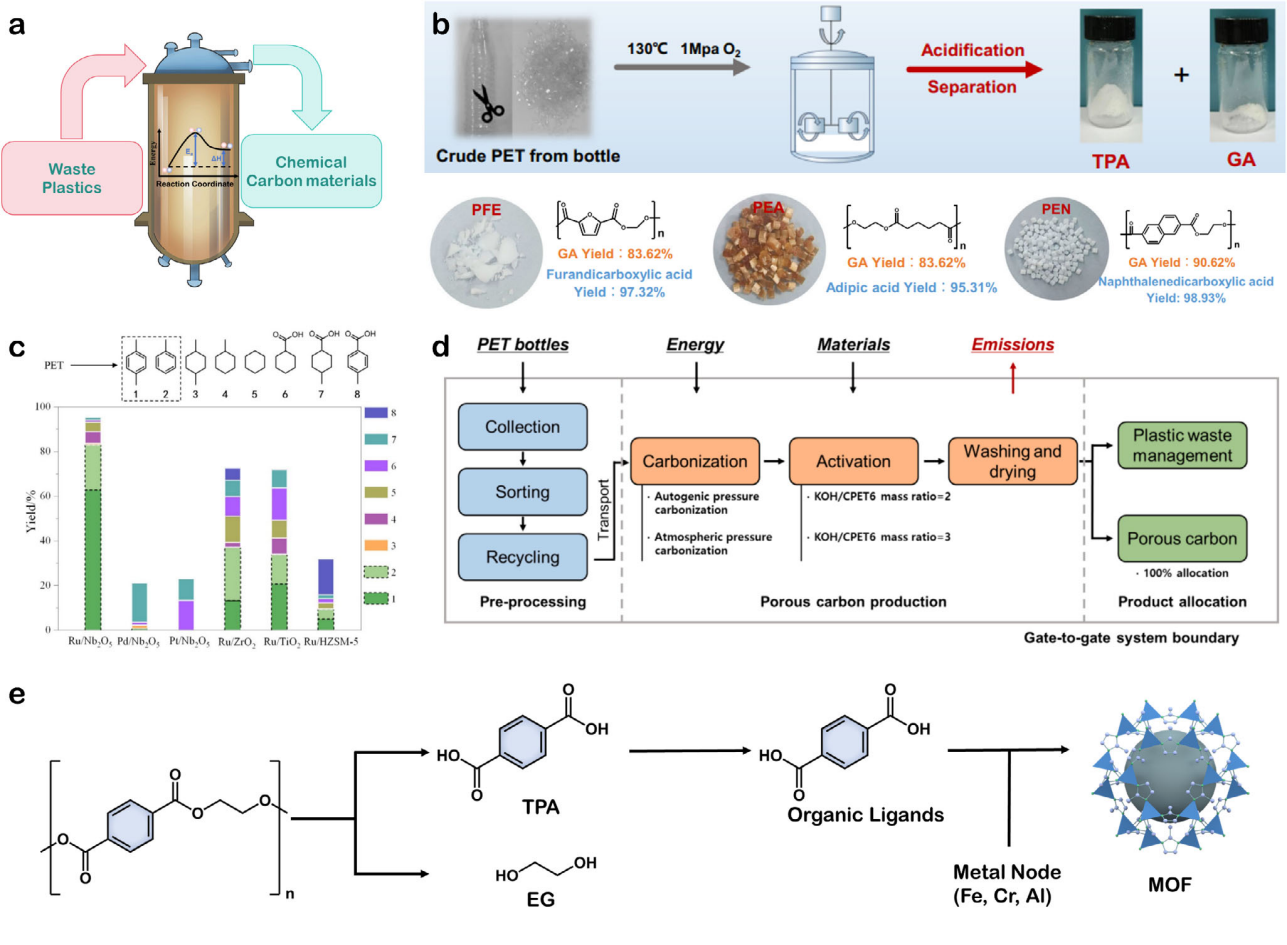
a) Schematic illustration of thermocatalytic upcycling. b) Upcycling of PET plastics and the subsequent separation and purification process of TPA and GA products. Reproduced with permission.^[^ ^134^ ^]^ Copyright 2024, Springer Nature. c) The conversion of PET over various catalysts. Reproduced with permission.^[^ ^135^ ^]^ Copyright 2021, Wiley. d) LCA of porous carbon production from waste PET bottles. Reproduced with permission.^[^ ^136^ ^]^ Copyright 2024, Elsevier. e) Schematic illustration of MOF material generated from PET.

Due to the intrinsic chemical inertness of polyester wastes, elevated temperatures and pressures are typically required to overcome the activation energy needed for breaking the polymer backbone.^[^ ^126^, ^127^ ^]^ Unlike conventional thermochemical solvent depolymerization methods that primarily recover building blocks from polyester wastes, thermocatalytic reforming achieves complete cleavage and reconstruction of polyester molecular chains. This often involves complex reaction pathways, including the cleavage of ester bonds, C*─*C bonds and even aromatic ring rearrangements. By this way, ester linkages within the polymer chains undergo scission, generating a range of high‐value small‐molecule chemicals and carbonaceous materials (**Figure** **8**a).^[^ ^128^, ^129^, ^130^, ^131^ ^]^ Plastic Energy company has developed a method to produce pyrolysis oil from postconsumer plastic waste.^[^ ^132^ ^]^ This technology has been validated at an industrial‐scale facility in Spain, employing an oxygen‐free pyrolysis process to convert mixed plastic waste into high‐quality feedstock suitable for the production of new plastics. To reduce the high energy consumption associated with thermal processes, tailored catalysts have been employed to lower activation energy and control product selectivity, thereby improving economic efficiency.^[^ ^133^ ^]^

Typically, thermocatalytic upcycling of polyester wastes generates a complex products mixture with a broad carbon‐number distribution. This broad product distribution arises from the random bond cleavage pathways under high‐temperature environment, initiating a cascade of secondary reactions, such as cracking, aromatization, and condensation. While this reactivity expands the range of potential chemical outputs, it also complicates downstream separation and purification due to the lack of product specificity. Therefore, rational catalyst design plays a key role in modulating reaction pathways, enhancing product specificity, and enabling efficient recovery of target chemicals. Chen et al. employed a gold‐decorated nickel oxide catalyst (Au/NiO) to selective upcycling PET into a high‐value of product glycolic acid (GA), achieving a GA yield of 87.6% under 130 °C and 1 MPa O_2_.^[^ ^134^ ^]^ In a one‐step thermocatalytic oxidation, oxygen vacancies on the Au/NiO catalyst facilitated both PET hydrolysis and subsequent oxidation reactions, while preferential TPA adsorption on NiO prevented active‐site blockage, enabling efficient conversion of EG to GA. Mechanistic analyses revealed that at the Au/NiO*─*O_v_ interface, EG molecules were activated and hydrogen abstraction occurred via lattice oxygen, generating glycolaldehyde intermediates that were further oxidized to GA by hydroxyl radicals. This synergistic pathway allowed efficient one‐step conversion of PET into TPA and GA, and was extendable to other polyester plastics, demonstrating both catalytic efficiency and economic viability (Figure 8b). Jing et al. developed a Ru/Nb_2_O_5_ catalyst that enabled selective cleavage of C*─*O and C*─*C bonds in PET using octane as the solvent under 280–320 °C and 0.5 MPa H_2_.^[^ ^135^ ^]^ The cooperative interaction between small Ru clusters and NbO*_x_* species suppressed hydrogenation of aromatic rings, leading to high selectivity toward benzene and toluene production (Figure 8c). Mi et al. employed a Zn‐modified copper catalyst (CuZnSi‐400) to convert PLA into 1,2‐propanediol (PDO) under solvent‐free conditions at 220 °C and 4 MPa H_2_, achieving a remarkable conversion and selectivity of 99.5%.^[^ ^137^ ^]^ Initially, molten PLA (above 160 °C) comes into contact with the catalyst, where a small fraction of ester bonds undergoes direct hydrogenolysis to produce an initial amount of PDO. The generated PDO acts as an in situ alcohol solvent, facilitating the alcoholysis of PLA into lactyl propylene glycol esters, which are subsequently and rapidly hydrogenolyzed into additional PDO. Moreover, the introduction of additional small‐molecule reactants can enable the production of coupled products in thermocatalytic upcycling of polyesters. Tian et al. demonstrated that a Ru/TiO_2_ catalyst in aqueous ammonia at 140 °C effectively converted PLA waste into alanine, a high‐value amino acid.^[^ ^138^ ^]^ Ru nanoparticles are employed to activate the α‐C*─*H bonds of the intermediate ammonium lactate, while TiO_2_ stabilizes the Ru particles through strong metal–support interactions. This synergistic effect enabled an alanine yield of 77% without external hydrogen input, featuring high selectivity, low energy demand, and excellent environmental compatibility.

Beyond chemical production, thermocatalytic reforming also provides an effective pathway for generating advanced carbon materials. Owing to their high hydrocarbon content, polyester plastics particularly petroleum‐based polyesters are ideal precursors for carbonaceous frameworks.^[^ ^139^ ^]^ During catalytic pyrolysis or graphitization, released carbon atoms reorganize into ordered structures, such as graphene and carbon nanotubes.^[^ ^140^ ^]^ Li et al. optimized PET carbonization in a sealed reactor at 600 °C to produce porous carbon materials (Figure 8d).^[^ ^136^ ^]^ Compared with conventional carbonization processes, which typically achieve carbon conversion rates of less than 20%, this study employed a sealed reactor that facilitated secondary reactions of pyrolysis products under high pressure, thereby increasing the carbon conversion rate to 50%. A LCA revealed that the global warming potential (GWP) of this carbonization process was 8.7 kg CO_2_ kg^−1^, significantly lower than traditional processes due to reduced energy use and emissions.

Additionally, polyester‐derived aromatic monomers, particularly TPA, can serve as ligand precursors to coordinate with metal ions for the synthesis of metal–organic frameworks (MOFs) (Figure 8e). Owing to their ultrahigh surface areas, tunable pore architectures, and modular functionality, MOFs have emerged as versatile platforms for applications in gas storage, catalysis, separation, and sensing. Recent studies have demonstrated the feasibility of synthesizing high‐performance MOFs directly from depolymerized PET.^[^ ^64^ ^]^ Yun et al. developed an efficient upcycling strategy in which PET wastes were fully depolymerized via neutral hydrolysis catalyzed by ultrasmall ZnO nanoparticles, achieving a TPA yield of 95.6% at 200 °C within 60 min.^[^ ^141^ ^]^ The resulting r‐TPA was subsequently employed to synthesize a series of MOFs, including MIL‐101(Cr), UiO‐66(Zr), UiO‐66(Ce), MIL‐53(Al), and MIL‐53(Al, Fe), via one‐pot or two‐pot protocols. These MOFs exhibited high crystallinity and surface areas comparable to, or even surpassing, those of their petroleum‐derived counterparts. Notably, the BET surface area of MIL‐101(Cr) reached 2591.9 m^2^ g^−1^, outperforming conventionally synthesized counterparts and underscoring the feasibility of transforming PET waste into high‐performance porous materials.

While thermocatalysis highlights the potential for integrating polyester waste valorization with the recovery of value‐added chemicals and functional carbonaceous materials. However, its practical implementation remains hindered by high thermal energy demands, substantial carbon emissions and limited product selectivity. Future research should therefore focus on developing low‐temperature and energy‐efficient catalytic systems capable of operating under milder conditions, along with catalysts featuring precisely tuned active sites that promote moderate interactions between polymers and catalysts. Such strategies will be crucial to enhance reaction specificity, suppress side reactions, and facilitate efficient product recovery, ultimately enabling thermocatalytic upcycling to contribute meaningfully to a circular and low‐carbon polyester economy.

### 4.2 Photocatalytic Upcycling of Polyester Plastic Wastes

The photocatalytic upcycling of polyester wastes essentially follows similar principles to traditional photocatalytic redox reactions and involves three fundamental steps: 1) light absorption by the photocatalyst and generation of electron–hole pairs, 2) effective separation and migration of these photogenerated charge carriers to the catalyst surface, and 3) surface redox reactions. The photogenerated carriers directly or indirectly interact with polymer molecules, resulting in their degradation and transformation.^[^ ^142^ ^]^ Two main mechanistic pathways have been identified: radical‐mediated mineralization and direct hole‐driven oxidation.^[^ ^143^, ^144^ ^]^ In the radical pathway, photogenerated holes and electrons react with H_2_O and O_2_ to generate reactive oxygen species (ROS), such as hydroxyl radicals (·OH) and superoxide anions (·O_2_^−^), which indiscriminately cleave C*─*C and C*─*H bonds in polymer chains. This often results in complete mineralization to CO_2_, which may subsequently undergo photoreduction to value‐added products such as FA or acetic acid (AA), offering a potential route to carbon valorization. This mechanism is especially effective for recalcitrant polyolefins, of which the chemical inertness limits selective depolymerization. In contrast, the direct oxidation pathway relies on photogenerated holes abstracting hydrogen atoms from electron‐rich sites along the polymer backbone, which particularly processes effectiveness in reforming of polar polyesters that contain ester, hydroxyl, or carboxyl functionalities. This approach enables selective cleavage of C*─*H and C*─*C bonds, affording intermediates such as EG, TPA, or LA, which can undergo further oxidative transformation into fuels or commodity chemicals.

**Figure 9:**
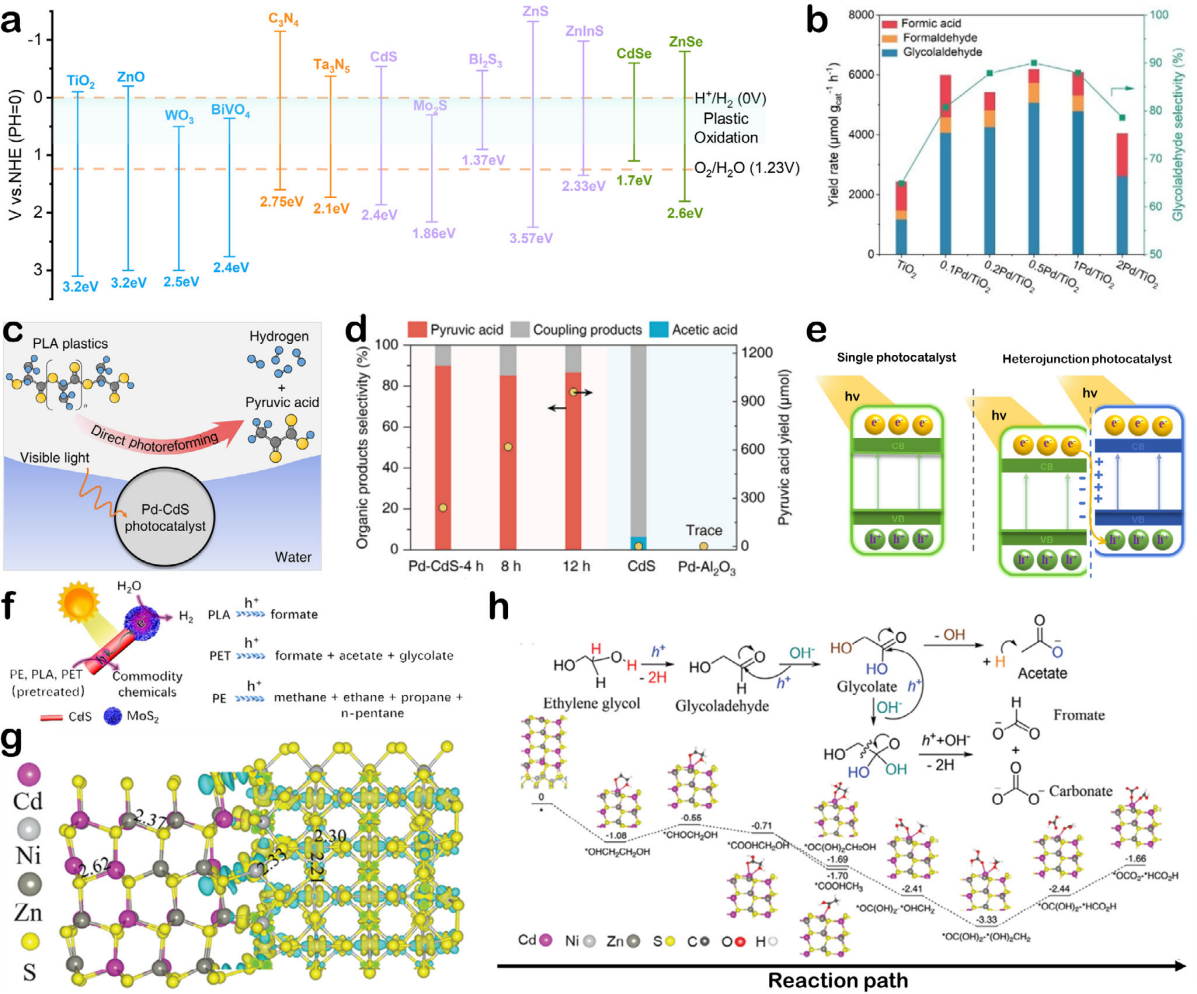
a) Band positions of commonly used semiconductor based photocatalysts. b) Product yield and glycolaldehyde selectivity of TiO_2_ and Pd/TiO_2_ in the photocatalytic EG oxidation reaction. Reproduced with permission.^[^ ^145^ ^]^ Copyright 2025, Wiley. c) Schematic illustration of the PLA photocatalytic upcycling process over Pd‐CdS photocatalyst. d) Product selectivity and yield of pyruvic acid over Pd‐CdS, CdS, and Pd‐Al_2_O_3_. Reproduced with permission.^[^ ^146^ ^]^ Copyright 2024, American Chemical Society. e) Schematic illustration of single photocatalysts and heterojunction photocatalysts. f) Photoreforming of plastic wastes schematic illustration for MoS_2_/CdS heterojunction photocatalysts. Reproduced with permission.^[^ ^147^ ^]^ Copyright 2022, American Chemical Society. g) Charge density difference plot of the N/ZCS model. h) EG degradation pathways and a degradation reaction path energy panel on the NS/ZCS model. Reproduced with permission.^[^ ^148^ ^]^ Copyright 2025, Wiley.

Early studies on photocatalytic upcycling drew parallels with photocatalytic water splitting, where polyester plastics functioned analogously to sacrificial agents. The overall efficiency of water splitting is constrained by the sluggish oxygen evolution reaction (OER), which require substantial kinetic overpotentials. Additionally, only about 43% of the solar spectrum possesses sufficient energy to drive this process.^[^ ^149^ ^]^ In contrast, the oxidation potentials required for plastic degradation are significantly lower than those for water oxidation (**Figure** **9**a). For instance, the Gibbs free energy changes associated with reforming the PET monomer EG and the PLA monomer LA are only +9.2 and +27 kJ mol^−1^, respectively. These values are considerably lower than +237 kJ mol^−1^ required for water splitting, indicating greater thermodynamic feasibility.^[^ ^150^ ^]^ Taylor et al. employed CdS/CdO*_x_* quantum dots as photocatalysts to upcycling PET, PLA, and polyurethane (PUR), achieving the generation of H_2_ and various organic products, including pyruvic acid (PA), FA, AA under alkaline aqueous conditions.^[^ ^151^ ^]^ Moreover, pretreating PET, PLA, and other polyesters under alkaline conditions to obtain oligomers or monomers facilitates their subsequent photoreforming. Pretreatment with 10 m NaOH markedly increased H_2_ yields from PET and PUR, resulting in significantly enhanced hydrogen production. To improve product selectivity and enhance reaction rates, researchers have made significant progress in tuning reaction pathways and optimizing operating conditions, laying a solid foundation for further advances in the photocatalytic upcycling of polyester plastic wastes.

Current photocatalyst employed for polyester wastes upcycling include metal‐based photocatalysts^[^ ^64^, ^152^, ^153^ ^]^ and metal free photocatalysts.^[^ ^154^ ^]^ Noble metals are widely applied in photocatalytic upcycling of polyester wastes due to their superior electron transfer capabilities and strong adsorption and activation properties, which significantly lower activation energies and accelerate the transformation of polyester plastics.^[^ ^155^ ^]^ Moreover, by tailoring their crystalline facets or alloying strategies, noble‐metal catalysts can precisely regulate reaction pathways and promote the formation of high‐value‐added products. They also exhibit high resistance to deactivation by acidic intermediates or radicals, maintaining stable performance over long‐term operation. Zhang et al. synthesized a Pd single‐atom catalyst anchored on TiO_2_ (Pd/TiO_2_) via a low‐temperature photochemical approach, which facilitated the highly selective oxidation of EG to acetaldehyde (selectivity >90%) (Figure 9b).^[^ ^145^ ^]^ Mechanistic studies have revealed that Pd sites attract and accumulate photogenerated holes, enhancing the participation of holes in oxidation reactions. The holes oxidize the hydroxyl groups of EG to generate ethoxy radicals, which serve as key intermediates for the formation of acetaldehyde while preventing overoxidation to undesired C_1_ products. Similarly, Miao et al. developed a Pd‐modified CdS (Pd‐CdS) catalyst for visible‐light‐driven decomposition of PLA (Figure 9c), achieving high H_2_ evolution and 95.9% selectivity toward pyruvate (PA) over 100 h of reaction (Figure 9d).^[^ ^146^ ^]^ Theoretical calculations indicate that Pd sites inhibit both lactate coupling and decarboxylation pathways, thereby preventing the formation of acetate and CO_2_. Additionally, PA exhibits weak adsorption on Pd‐CdS, allowing for timely desorption and effectively avoiding overoxidation. Considering into the cost and environmental concerns associated with metal‐based catalysts, recent research has shifted toward developing sustainable, low‐cost alternatives aligned with green chemistry and circular economy principles.

Heterojunction photocatalysts, constructed by coupling two or more semiconductors (or semiconductors and metals), exhibit enhanced interfacial charge separation efficiency and tunable band structures. These heterostructures utilize interfacial band engineering, including band bending and band alignment, and built‐in electric fields to promote efficient spatial separation of photogenerated electron–hole pairs, effectively mitigating charge recombination that limits single‐semiconductor systems (Figure 9e).^[^ ^156^ ^]^ Du et al. developed MoS_2_‐modified CdS nanorod heterojunctions (MoS_2_/CdS) to catalyze the transformation of PLA and PET into high‐value products and H_2_ (Figure 9f).^[^ ^147^ ^]^ The results showed that PLA was oxidized to FA (5.37 mmol L^−1^) and carbonate, while PET yielded FA, AA, and GA. MoS_2_ traps electrons and accelerates their transfer from CdS, while also promoting H_2_ evolution by lowering the overpotential. Oxidative holes on CdS directly attack plastic molecules, with directional charge separation from heterojunction driving the high efficiency. Ma et al. reported a Ni_3_S_4_/ZnCdS (NS/ZCS) heterojunction system that efficiently reformed PLA and PET into clean hydrogen and valuable chemicals.^[^ ^148^ ^]^ PLA was predominantly converted to PA with 94.2% selectivity, while PET was transformed into AA with 78.3% selectivity, achieving carbon yields of 26.5% and 2.24%, respectively. Bader charge analysis confirmed that electrons are transferred from ZCS to Ni_3_S_4_, indicating that Ni_3_S_4_ plays a crucial role in constructing an electron transfer channel, thereby promoting effective separation of photogenerated electron–hole pairs (Figure 9g). Moreover, the energy barriers of key steps such as dehydrogenation during the degradation of EG on the NS/ZCS are significantly lower than those on the pure ZCS by theoretical calculations, which explains its enhanced catalytic activity (Figure 9h). Through compositional design and interface engineering, heterojunction photocatalysts strike a promising balance among activity, stability and cost, making them highly attractive for plastic upcycling.^[^ ^157^ ^]^ Future breakthroughs are likely to stem from the integration of interfacial modulation at the atomic scale with machine learning assisted materials design.

Currently, most carbon‐based photocatalysts for plastic upcycling are derived from graphitic carbon nitride (g‐C*_x_* N*_y_*), owing to its suitable bandgap, chemical robustness and low‐cost precursors.^[^ ^158^ ^]^ Han et al. developed a metal‐free composite photocatalyst composed of carbonized polymer dots and graphitic carbon nitride (CPDs‐CN), which enabled efficient oxidation of EG to GA and AA, while simultaneously achieving a high H_2_ production rate of 1034 µmol g^−1^ h^−1^.^[^ ^159^ ^]^ Owing to its unique physicochemical properties, g‐C*_x_* N*_y_* presents broad prospects in plastic valorization. Future research may focus on material modification and process engineering, including the incorporation of metal‐based cocatalysts or heterojunction formation to enhance light harvesting, improve charge separation and regulate reaction pathways for higher product selectivity.

### 4.3 Electrocatalytic Upcycling of Polyester Plastics Waste

Electrocatalysis, powered by renewable electricity, enables controllable redox transformations through directional electron transfer, facilitating the generation of key intermediates on electrode surfaces and guiding the selective conversion of reactants into value‐added products.^[^ ^160^, ^161^ ^]^ For polyester waste streams, a hydrolytic pretreatment step is typically employed to generate soluble oligomers or monomers, thereby overcoming the kinetic limitations imposed by heterogeneous solid–liquid interfaces.^[^ ^66^ ^]^ Compared to photocatalytic approaches, electrocatalysis offers improved selectivity and efficiency, attributed to its tunable redox potentials and precise control over electron‐transfer pathways.^[^ ^162^, ^163^ ^]^ These advantages have spurred increased interest in applying electrocatalysis to polyester wastes valorization. Recent developments in this field have focused on adjustment of product selectivity, in situ identification of reactive intermediates and mechanistic elucidation.

**Figure 10:**
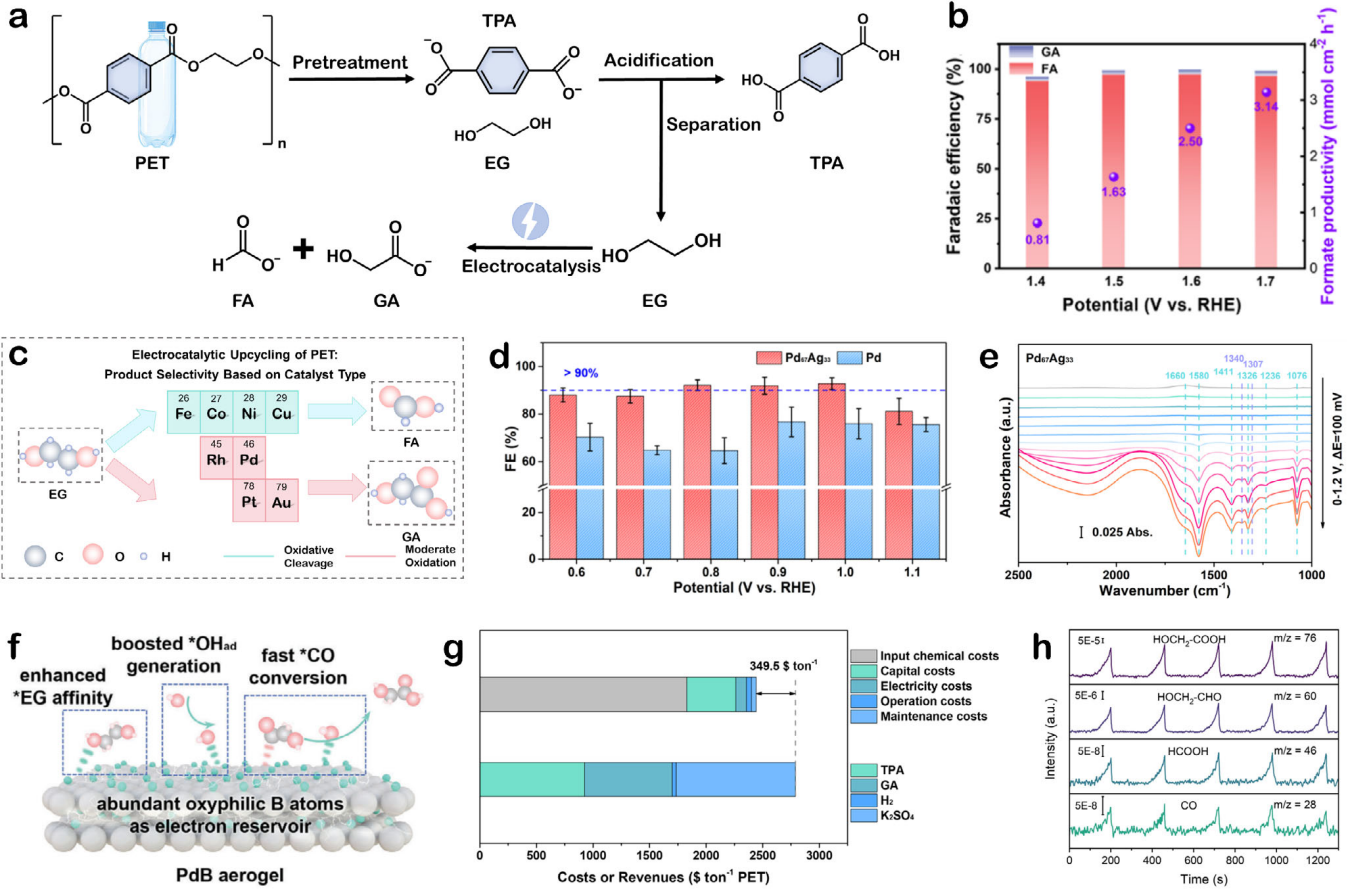
a) Schematic illustration of the electrocatalytic upgrading and recycling process for PET plastic wastes. b) FA productivity and FE for FA and GA. Reproduced with permission.^[^ ^164^ ^]^ Copyright 2025, American Chemical Society. c) Reaction route for electrocatalytic EG oxidation at different catalyst type. d) Potential dependence of FE for GA production on Pd and Pd_67_Ag_33_. e) In situ electrochemical infrared spectra for the EG oxidation reaction (EGOR) of moderate oxidation to generate GA. Reproduced with permission.^[^ ^165^ ^]^ Copyright 2024, PNAS. f) The EG to GA conversion process on PdB. g) TEA of electrocatalytic GA synthesis from PET wastes. Reproduced with permission.^[^ ^166^ ^]^ Copyright 2024, Wiley. h) DEMS of (PtIr)(FeMoBi). Reproduced with permission.^[^ ^167^ ^]^ Copyright 2025, Wiley.

Among polyester substrates, PET has emerged as a prototypical target due to its well‐defined depolymerization products (i.e., EG and TPA), which are amenable to downstream electrochemical conversion. Under acidic hydrolysis conditions, TPA precipitates and is readily isolated by filtration, while EG remains in solution and undergoes electro‐oxidation on the anode (**Figure** **10**a). Given EG's high boiling point (197.6 °C) and high water solubility, the selective electrochemical oxidation of PET‐derived EG into value‐added products offers a more energy‐efficient and economically favorable alternative to conventional separation strategies. Benefiting from progress in organic electrosynthesis, EG can be selectively converted into high‐value products, such as FA, GA, and potassium diformate (KDF) across various catalyst systems. ^[^ ^168^, ^169^, ^170^, ^171^ ^]^ Two dominant mechanistic pathways for EG oxidation have been proposed: i) C*─*C bond cleavage pathway yielding FA, and ii) C_2_ backbone perseveration leading to partial oxidation into GA.

For the C*─*C cleavage pathway, transition metal‐based catalysts usually exhibit strong adsorption and weak desorption toward reaction intermediates, favoring C*─*C bond activation and cleavage. Zhou et al. reported a Ni‐modified cobalt phosphide (CoNi_0.25_P) electrocatalyst capable of selectively oxidizing EG to FA with over 80% Faraday efficiency (FE) at 500 mA cm^−2^ in a membrane electrode assembly reactor.^[^ ^171^ ^]^ Moreover, the generated FA was employed as an acidifying agent for further TPA separation and conversion into KDF. TEA indicates that at a commercially relevant current density of 300 mA cm^−2^, upcycling 1 ton of waste PET can generate a net profit of ≈$8350. The increase in net profit is closely correlated with higher current densities, as elevated space‐time yields significantly reduce unit costs. Therefore, developing catalytic systems capable of sustaining high current densities is critical for scaling practical applications. Sun et al. designed a heterostructured catalyst composed of crystalline Ni_3_S_2_ and amorphous CoNiOOH for upcycling PET wastes.^[^ ^164^ ^]^ As shown in Figure 10b, the FE of FA remained around 95.0% even at high current densities exceeding 500 mA cm^−2^ across different applied potentials. The amorphous/crystalline interface induces an upward shift of the *d*‐band center, which enhances the adsorption of ·OH and EG, thereby promoting C*─*C bond cleavage and consecutive dehydrogenation of EG. On this basis, a series of catalysts have been developed that maintain FE above 90% across a wide range of current densities from 100 to 500 mA cm^−2^ (**Table** **3**).^[^ ^172^, ^173^ ^]^

**Table 3:** Summary of different electrocatalysts reported for PET and PLA upcycling.

Plastic	Catalyst	Production	Current density [mA cm^−2^]	FE [%]
PET	CoNi_0.5_P^[^ ^171^ ^]^	FA (KDF)	500	80
	AC‐NiO^[^ ^168^ ^]^	FA	1000	80
	Ni_0.3_Co_0.7_/IF^[^ ^172^ ^]^	FA	100	90
	Mo‐Ni(OH)_2_^[^ ^173^ ^]^	FA	300	96
	CoNiOOH‐Ni_3_S_2_/NF^[^ ^164^ ^]^	FA	500	97
	LM‐PdCu^[^ ^174^ ^]^	GA	50	92
	Pd_67_Ag_33_^[^ ^165^ ^]^	GA	300	93
	PdB^[^ ^166^ ^]^	GA	170	94
	(PtIr)(FeMoBi)^[^ ^167^ ^]^	GA	200	95
	Pd/NiMoO_4_/NF^[^ ^170^ ^]^	GA	230	99
PLA	Ni_2_P/NF^[^ ^175^ ^]^	AA	50	92
	NiSe_2_^[^ ^176^ ^]^	AA	100	95
	Ni(Co)OOH^[^ ^177^ ^]^	AA	400	96
	CoSe_2_/NF^[^ ^178^ ^]^	AA	100	97
	Pd/Ni(OH)_2_/NF^[^ ^179^ ^]^	PA	10	90

Compared to FA, GA has higher commercial value due to its broad applications in biodegradable polyesters (e.g., PGA) and high‐end cosmetics, making it a more desirable product.^[^ ^180^ ^]^ However, achieving high GA selectivity remains more challenging than FA. Efficient GA production requires precise potential control to oxidize only one hydroxyl group into a carboxylic acid while retaining the other and avoiding C*─*C bond cleavage. This desirable pathway involves only a four‐electron (4 e^−^) transfer, resulting in lower energy consumption. Nevertheless, terminating the reaction at the GA stage and preventing its overoxidation to FA remains a major technical challenge. Noble metal‐based catalysts such as Pd show promise in suppressing overoxidation due to their unique electronic properties. As shown in Table 3, transition‐metal‐based catalysts generally facilitate C*─*C bond cleavage leading to FA, whereas noble‐metal catalysts favor partial oxidation, yielding GA (Figure 10c). In this regard, our group developed a PdAg alloy aerogel catalyst.^[^ ^165^ ^]^ The alloying strategy with Ag enables controlled modulation of morphology, electronic structure, and catalytic performance. Alloying with Ag introduced ligand and lattice strain effects, which modulated the EG oxidation pathway and significantly enhanced GA selectivity. In addition, the Ag incorporation lowered the *d*‐band center of Pd, weakening intermediate adsorption and suppressing C*─*C cleavage and C_1_ product formation. The catalyst Pd_67_Ag_33_ achieved a maximum FE for GA of 92.7% at 1.0 V versus RHE, which is significantly higher than that of pure Pd (Figure 10d). In situ infrared spectra confirmed this behavior (Figure 10e), with intensified GA‐specific peaks and no significant increase in FA signal, indicating successful pathway tuning via alloying.

Subsequently, our group identified that excessive adsorption of the ·COCH_2_OH intermediate led to C*─*C bond cleavage and C_1_ product formation by theoretical calculations (Figure 10f).^[^ ^166^ ^]^ Adjusting the catalyst's electronic structure, especially by lowering the *d*‐band center, effectively reduced the adsorption strength of intermediates and facilitated the desorption of C_2_ products. This insight guided the design of Pd‐based catalysts via strategies such as boron doping and Ag alloying, offering mechanistic understanding and practical routes for selective PET upcycling. Furthermore, it was estimated that at a current density of 200 mA cm^−2^ (0.9 V vs RHE), the revenue generated from converting one ton of PET into GA would be ≈$349.5, demonstrating the economic potential of this process (Figure 10g). Based on this, Hao et al. synthesized an L1_0_‐(PtIr)(FeMoBi) high‐entropy intermetallic catalyst via a lattice‐compensation strategy.^[^ ^167^ ^]^ The Pt/Ir sites preferentially adsorbed EG, while Fe/Mo sites facilitated ·OH adsorption, enabling efficient EG to GA conversion and effective suppression of C*─*C bond cleavage. Differential electrochemical mass spectrometry (DEMS) analysis showed that the intensity of C_2_ intermediates exceeded that of C_1_ species by three orders of magnitude (Figure 10h), further confirming the dominance of the EG—glycolaldehyde—GA pathway with high C_2_ selectivity.

**Figure 11:**
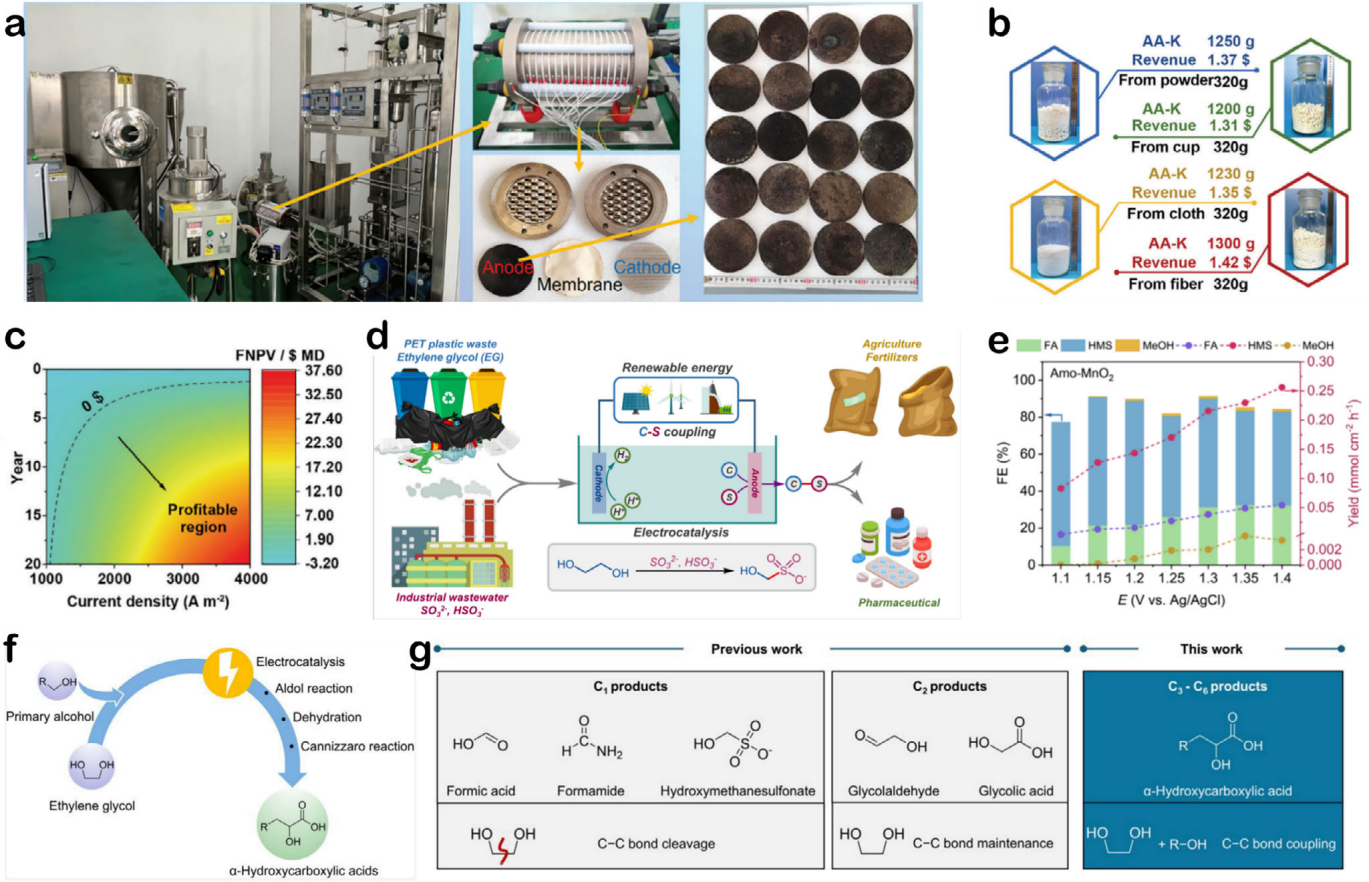
a) Performance of the industrial‐scale electrolysis device. b) Solid AA‐K derived from various actual PLA plastic waste sources by electrocatalysis upcycling. c) Economic profitability and cost analysis of the industrial‐grade system. Reproduced with permission.^[^ ^177^ ^]^ Copyright 2025, Wiley. d) Schematic diagram of electrocatalytic PET upcycling to HMS. e) FE and yield of products at 1.1–1.4 V (vs Ag/AgCl) ranging with Amo‐MnO_2_ as electrocatalysis. Reproduced with permission.^[^ ^181^ ^]^ Copyright 2024, American Chemical Society. f) Schematic illustration of PET upcycling to LA. g) Previous work on EG oxidation to C_1_ or C_2_ products and this work on EG and alcohol co‐oxidation to C_3_‐C_6_ α‐HCAs. Reproduced with permission.^[^ ^182^ ^]^ Copyright 2025, American Chemical Society.

The electrocatalytic upcycling of bio‐based polyester PLA has also gained notable attention. The depolymerization of PLA under natural conditions proceeds extremely slowly, potentially leading to greenhouse gas emissions and microplastic accumulation. Therefore, developing suitable electrocatalytic systems to convert PLA into high‐value‐added products represents a promising strategy to mitigate these environmental challenges. Unlike PET, whose depolymerization yields two distinct products, PLA directly produces a single monomer LA, enabling simplified downstream electrocatalytic conversion without additional separation steps. Hu et al. developed a Ni‐based electrocatalyst Ni(Co)OOH by cobalt (Co) doping of NiOOH, and applied it for the upcycling of PLA into potassium acetate (AA‐K).^[^ ^177^ ^]^ Under an applied potential of 1.40 V versus RHE, the system achieved a current density of 403 mA cm^−2^ and a FE of 96% for AA‐K production. Notably, the authors integrated this reaction into a scalable industrial process, encompassing steps such as KOH pretreatment of PLA waste, electrooxidation of LA to AA, pH adjustment using AA and spray drying to obtain solid AA‐K (**Figure** **11**a). Mechanistic studies revealed that LA‐K first adsorbs onto the active sites of the NiCo catalyst. Subsequently, the α‐hydrogen is removed to form a reaction intermediate potassium pyruvate (PA‐K), which then undergoes bond cleavage to generate AA‐K. Various real‐world PLA plastic wastes were effectively converted using this system and achieving high conversion efficiencies (Figure 11b). Economic analysis further demonstrated a net profit of $542 per ton of PLA treated, an annual profit of $1.04 million at a current density of 2000 A m^−2^, and a payback period of 4 years (Figure 11c). This work provides a potentially scalable and carbon‐neutral pathway for the valorization of PLA waste. Similar to PET, the electrocatalytic transformation of PLA involves oxidative bond cleavage and decarboxylation. Among the oxidation products, PA‐K (a C_3_ compound) possesses significantly higher value than AA‐K (a C_2_ product) (Table 3). However, only a limited number of studies have reported the formation of PA‐K via electrocatalytic routes.^[^ ^179^ ^]^ Future research should prioritize the rational design of electrocatalysts to selectively promote the formation of C_3_ products such as PA while minimizing C_2_ by‐products, thereby improving the economic feasibility of PLA electrocatalytic upcycling.

Beyond conventional oxidation reactions, recent efforts have focused on coupling electrocatalytic oxidation with coupling reactions to broaden the product scope of PET waste. For example, Kang et al. employed an amorphous MnO_2_ catalyst to couple EG with sulfite under near‐neutral conditions (Figure 11d), producing hydroxymethanesulfonate with a FE of 70% (Figure 11e).^[^ ^181^ ^]^ In a neutral electrolyte, EG is oxidized on the amorphous MnO_2_ anode to generate the electrophilic intermediate ·CH_2_OH, which subsequently couples with sulfur‐based nucleophiles to form the target product. Shi et al. developed a tungsten oxide catalyst for the electrocatalytic amination of EG with ammonia (NH_3_), yielding formamide (HCONH_2_) via an EG to glyoxal intermediate and C*─*N bond formation with NH_3_‐derived N‐radicals.^[^ ^183^ ^]^ Specifically, EG and NH_3_ are oxidized on the WO_3_ surface to generate adsorbed glycolaldehyde (·OCH_2_CHO) and ·NH_2_ species, respectively. The ·NH_2_ acts as a nucleophile, attacking glycolaldehyde and inducing C*─*C bond cleavage to produce HCONH_2_ and formaldehyde (HCHO). The resulting HCHO is further attacked by ·NH_2_ to produce an additional molecule of HCONH_2_, achieving complete carbon utilization. Subsequently, the same group achieved electrocatalytic cross‐coupling of EG and methanol to synthesize C_3_ LA with a productivity of 268.1 µmol cm^−2^ h^−1^(Figure 11f).^[^ ^182^ ^]^ This approach was later extended to generate longer‐chain C_4_‐C_6_ α‐hydroxycarboxylic acids (α‐HCAs) using ethanol and propanol as feedstocks (Figure 11g). These studies demonstrate the versatility of electrocatalytic coupling strategies in forming C*─*S, C*─*N, and C*─*C bonds, offering new opportunities for diversifying the chemical valorization of plastic‐derived monomers.

Given that electrocatalytic upcycling of polyester wastes predominantly proceeds at the anode, increasing attention has been directed toward the strategic coupling of value‐added cathodic reactions. In conventional water electrolysis, the anodic OER suffers from sluggish kinetics and a high overpotential, leading to high energy demands and the generation of low‐value O_2_. Replacing OER with plastic oxidation thus presents an opportunity to lower the system's energy input while simultaneously valorizing both half‐reactions. Li et al. reported a bifunctional electrocatalytic system employing metallic nickel (Ni), which enables simultaneous electrocatalytic upcycling of PET waste at the anode and efficient hydrogen production at the cathode within the same electrolyzer.^[^ ^169^ ^]^ Under anodic conditions, Ni undergoes in situ reconstruction into the active NiOOH phase, which selectively oxidizes EG into FA via a chemical oxidation pathway, achieving a FE of 90% at 1.5 V versus RHE. Meanwhile, at the cathode, a thin layer of amorphous Ni(OH)_2_ forms on the Ni surface during the hydrogen evolution reaction (HER), generating a Ni/Ni(OH)_2_ core‐shell structure with a low overpotential of 250 mV at 100 mA cm^−2^ and nearly 100% FE for hydrogen production. This catalytic system demonstrates excellent durability, maintaining stable operation in a flow cell for over 720 h. Compared to the conventional OER||HER electrolyzer, the integrated EGOR||HER system reduces the required cell potential by 350 mV at 100 mA cm^−2^, highlighting significant energy savings and enhanced industrial feasibility. Beyond HER, nitrate reduction (NO_3_RR) at the cathode has also been explored to pair with anodic plastic upcycling. Nitrate is a common nitrogen pollutant that poses health risks after conversion to nitrite in the human body. Electrochemical reduction offers an efficient route to convert nitrate into nitrogen gas or valuable amines, mitigating water pollution and improve drinking water safety.^[^ ^184^, ^185^ ^]^ Wu et al. developed a Cu@CoCu LDH/CC heterostructured nanosheet electrode for a dual‐function electrolyzer coupling nitrate wastewater reduction to NH_3_ at the cathode with PET upcycling at the anode.^[^ ^186^ ^]^ The EGOR||NO_3_RR electrolyzer exhibited outstanding performance, achieving 98.6% FE for NH_3_ and 98.1% for formate at a cell voltage of 1.3 V, offering 12% energy savings compared to the traditional OER||NO_3_RR system. Collectively, these coupled electrocatalytic strategies exhibit remarkable potential and multiple advantages, establishing them as promising approaches for advancing plastic waste valorization. With further research and technological advances, such coupling approaches are expected to offer innovative solutions for sustainable energy and chemical production.

Despite the significant progress achieved in electrocatalytic upcycling of polyester wastes, several challenges remain. Nonpolar and chemically inert polyolefin plastics remain largely inaccessible to current electrocatalytic methods. Moreover, the need for hydrolysis pretreatment in most systems increases process complexity and operational cost. Future research should therefore focus on developing highly active electrocatalysts with tunable electronic structures and enhanced interfacial reactivity to overcome limitations such as low conductivity and slow mass transfer constraints. In addition, coupling electrocatalysis with selective bond‐forming strategies offers a promising route toward multifunctional and precise plastic upcycling.

### 4.4 Biological Upcycling of Polyester Plastics Waste

Biotechnology represents another promising and sustainable strategy for plastic upcycling, enabling the production of a wide range of value‐added products. Engineered microorganisms can utilize depolymerized plastic monomers as carbon and energy sources and convert them into higher‐value compounds through intricate metabolic networks in bioreactors (**Table** **4**). For instance, EG can enter cellular metabolism via glycolaldehyde or acetaldehyde intermediates, eventually being transformed into acetyl‐CoA or PA and subsequently into amino acids, organic acids or polymeric materials.^[^ ^187^ ^]^ Panda et al. engineered *Escherichia coli* to efficiently produce the high‐value amino acid L‐tyrosine from EG.^[^ ^188^ ^]^ By overexpressing an oxygen‐tolerant mutant of *fucO* together with *aldA*, the strain showed enhanced EG utilization, producing 2 g L^−1^ of L‐tyrosine from 10 g L^−1^ EG, notably higher than the 1.2 g L^−1^ obtained from glucose under identical conditions. Kim et al. employed *Gluconobacter oxydans* to upcycle EG derived from PET hydrolysis into GA.^[^ ^189^ ^]^ Although many bacteria are known to utilize EG, the specific enzymatic mechanisms involved remain unclear. Ren et al. elucidated the metabolic pathway underlying the growth of *Paracoccus denitrificans* on EG, demonstrating that NAD‐dependent dehydrogenases mediate its efficient assimilation.^[^ ^190^ ^]^ The identified gene cluster (EtgR, EtgB, EtgA, and EtgC) was strongly induced by EG, with EtgB converting EG to glycolaldehyde and EtgA oxidizing it further to glycolate, while EtgR served as a transcriptional activator. Comprehensive structural, proteomic and adaptive evolution analyses confirmed the critical roles of these enzymes in EG metabolism, providing valuable insights and enzymatic tools for PET‐derived EG biotransformation and advancing microbial plastic upcycling.

**Table 4:** Summary of enzymes used for the biological upcycling of plastic monomers.

Plastic monomer	Enzyme/microorganism	Condition	Substrate
EG	*Ideonella sakaiensis* ^[^ ^189^ ^]^	40 °C PH = 8.0	Protocatechuic Acid, GA
	*E. coli* MG1655(DE3)^[^ ^188^ ^]^	30 °C PH = 7.0	L‐tyrosine
	*Paracoccus denitrificans* DSM 413^[^ ^190^ ^]^	30 °C PH = 7.5‐10	GA
	*Pseudomonas putida* KT2440^[^ ^191^ ^]^	30 °C PH = 7	PHA
TPA	*E. coli* GA^[^ ^192^ ^]^	30 °C PH = 7	Gallic acid
	*E. coli* BW25113ΔpabB^[^ ^193^ ^]^	37–50 °C PH = 7.4–8.0	Paracetamol
LA	*E. coli* BW25113^[^ ^194^ ^]^	37 °C PH = 7	1,2‐propanediol
	*Lactobacillus buchneri, L. parabuchneri* ^[^ ^195^ ^]^	20–30 °C PH = 3.8–5.8	1,2‐propanediol
	*E. coli* BL21(DE3) Codon Plus RP^[^ ^196^ ^]^	37 °C PH = n.r.	Acrylic acid
	*E. coli* Rosetta‐gami^[^ ^197^ ^]^	37 °C PH = 7	Propanol

Besides EG, TPA from another PET monomer can also be biologically upcycled. TPA is first transformed into TPA‐1,2‐cis‐dihydrodiol (DCD) by TPA 1,2‐dioxygenase (TphAabc), followed by oxidation with TPA‐1,2‐*cis*‐dihydrodiol dehydrogenase (TphB) to yield protocatechuic acid (PCA). As a critical intermediate, PCA can be further converted into valuable chemicals such as vanillin, gallic acid, catechol, muconic acid, and adipic acid.^[^ ^187^ ^]^ Recently, Johnson et al. developed a biocompatible Lossen rearrangement reaction in *Escherichia coli*, demonstrating that PET‐derived TPA can be converted into para‐aminobenzoic acid (PABA) and funneled through engineered pathways to produce the analgesic paracetamol.^[^ ^193^ ^]^ This work integrates nonenzymatic chemistry with cellular metabolism, establishing a novel chemo‐biological cascade for transforming plastic waste into high‐value pharmaceuticals and highlighting the potential of hybrid chemo‐bio systems in sustainable synthesis.

Since the major depolymerization product of PLA is LA, it typically functions as a terminal metabolite in cellular metabolism rather than as a common synthetic intermediate. Many microorganisms convert sugars into LA, but only a few are capable of using LA as a precursor for synthesizing more complex chemicals. Therefore, achieving PLA upcycling requires extensive metabolic engineering to efficiently channel LA into biosynthetic pathways, which is more challenging than in the case of PET. Moreover, high concentrations of LA can inhibit microbial growth and reduce biotransformation efficiency. Recently, several studies have reported the microbial conversion of LA into high‐value chemicals such as 1,2‐propanediol, acrylic acid and propanol via engineered biosynthetic pathways.^[^ ^196^, ^197^, ^198^ ^]^ Yesudhas et al. constructed a synthetic acrylate pathway in metabolically engineered *E. coli* to convert D‐LA and glucose into propionic acid.^[^ ^196^ ^]^ They screened and optimized key enzymes (Pct, Acr, and Lcd) from diverse microbial sources, and employed temperature induction and osmoprotectant strategies to improve enzyme solubility and activity. As a result, they achieved a propionic acid titer of 240–320 mg L^−1^ with a conversion rate of 32% from D‐LA, representing the highest reported level for propionic acid production via the acrylate pathway. This study demonstrated that enzyme source optimization and protein‐folding regulation can effectively overcome metabolic bottlenecks, offering new insights into the biological upcycling of PLA.

Overall, the biological upcycling of polyester plastic wastes offers multiple valorization routes, enabling the transformation of depolymerized intermediates into amino acids, organic acids, aromatics, and even pharmaceuticals. The integration of metabolic engineering with chemical strategies further expanded the product spectrum and demonstrated the potential of chemo‐bio hybrid pathways. Nevertheless, challenges remain, including low efficiency, narrow product scope, and limited microbial tolerance. Future advances will depend on identifying new metabolic nodes and key enzymes, as well as engineering robust microbial hosts with enhanced stress resistance and catalytic efficiency. Through these efforts, biological upcycling is expected to evolve into an essential component of a sustainable and circular plastic recycling system.

## 5 Conclusions and Perspective

**Figure 12:**
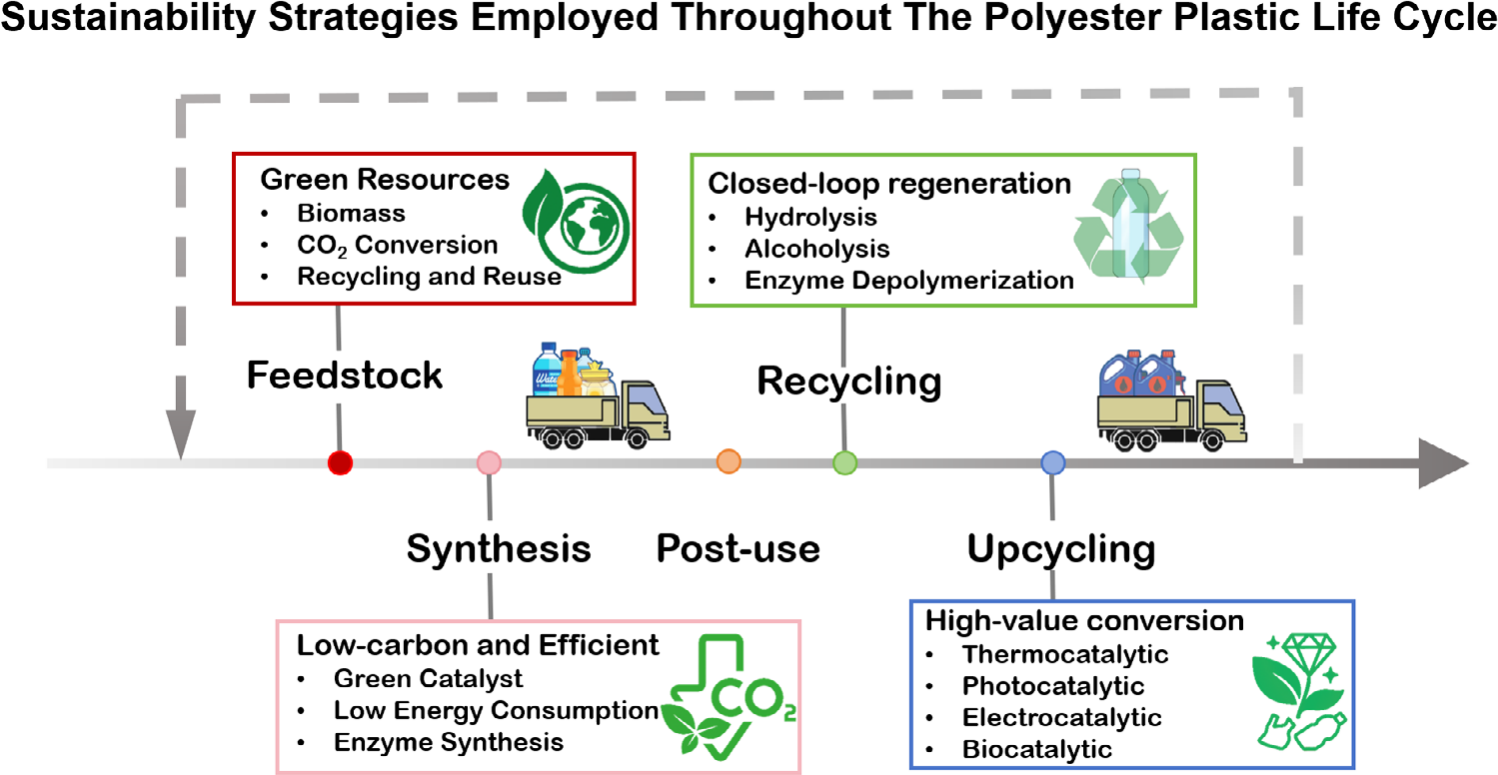
Sustainability strategies employed throughout the polyester plastic life cycle.

In response to mounting pressures from plastic pollution and carbon emissions, increasing attention has been directed toward the green synthesis and recycling of polyester plastics. This review systematically summarized recent advances and current challenges in the areas of sustainable synthesis, efficient recycling and value‐added upcycling of polyester‐based materials (**Figure** **12**). In terms of green synthesis, environmentally friendly and renewable pathways as well as various bio raw chemicals have been progressively developed by employing nontoxic catalysts, replacing fossil‐based feedstocks with renewable biomass or CO_2_, and integrating enzyme‐catalyzed polymerization processes. For effective recycling, conventional mechanical recycling still offers cost advantages, especially when combined with advanced sorting technologies, but often results in material property degradation. In contrast, chemical depolymerization methods such as hydrolysis and alcoholysis, along with enzymatic approaches, enable efficient molecular‐level closed‐loop recycling of polyesters. Coupling depolymerization with other chemical reactions can also yield a broader range of products beyond monomers. In the domain of upcycling, thermocatalysis, photocatalysis, electrocatalysis, and biological technology have opened new avenues for converting polyester waste into high‐value chemicals and fuels.

Despite notable progress across the life cycle of polyester plastics, several key challenges persist. Upcycling technologies are often limited to specific plastic types, such as pure PET, while real‐world plastic waste streams are typically heterogeneous, making it difficult to meet the demands of continuous processing. The presence of additives, such as plasticizers, further complicates the purification of recycled materials. Moreover, catalyst stability during recycling remains insufficient, degradation products are underutilized, and many emerging technologies suffer from poor scalability. Industrial chemical recycling facilities also require substantial capital investments, which can often reach several hundred million dollars, and the lack of standardized technological pathways further contributes to low industry adoption. Importantly, most current strategies fail to achieve a coherent integration of plastic synthesis and recycling. To address these limitations, future research should prioritize several key directions:

1. Recyclability of polyester plastics is progressively shifted from a “postconsumption remedy” to “design‐for‐recycling” at the source. Current technological innovations primarily focus on the systematic optimization of material formulations and structural design. For petroleum‐based polyesters, the incorporation of compatibilizers into virgin materials can enhance intermolecular interactions during recycling, thereby reducing performance degradation and extending the number of reuse cycles. For bio‐based polyesters, strategies have focused on incorporating degradable units or functional enzymes during polymerization to enable efficient depolymerization after use. Through source‐oriented design, it is expected that plastics can be prepared for circular regeneration from the outset of their production.
2. In the field of polyester wastes recycling, catalyst design still faces several critical challenges. Photocatalysts often suffer from high charge carrier recombination rates and poor stability, with many studies relying on CdS‐based materials that pose potential environmental risks and limit practical applications. Thermocatalysis and electrocatalysis commonly rely on noble metal catalysts, which are constrained by resource scarcity and high costs. Biological catalysis faces limitations such as insufficient activity, high costs, and limited stability. Therefore, it is essential to develop catalytic materials and systems that combine high activity, excellent selectivity, and environmental compatibility. Achieving a balance between environmental and economic benefits will be key to advancing plastic recycling technologies from laboratory research to industrial application. Moreover, advances in situ characterization techniques and computational simulations are essential for investigating reaction intermediates.
3. With the deepening understanding of reaction intermediates and mechanisms, cross‐coupling between reactive intermediates and introduced organic molecules has emerged as a promising strategy to expand product diversity. By rationally selecting specific intermediates and coupling reactants, it is possible to precisely construct key chemical bonds and functional groups such as C*─*C, C*─*N, C*─*O, and C*─*S, while minimizing by‐product formation and achieving both high atom economy and step economy. The resulting diverse portfolio of high‐value chemicals can be further utilized in the development of functional materials and advanced chemical products, offering new pathways for the industrialization of green synthesis technologies.
4. In recent years, thermocatalysis, photocatalysis, electrocatalysis, and biological technology have all made rapid progress in the field of catalytic plastic recycling. However, each individual approach has inherent limitations. Thermocatalysis typically requires high temperatures (above 200 °C), resulting in high energy consumption and a tendency toward side reactions such as coking or excessive cracking, which can lead to catalyst deactivation. Photocatalysis is constrained by the low solar energy flux and is affected by day‐night cycles and weather conditions, limiting overall light utilization efficiency. Electrocatalysis usually requires pretreatment of polyester plastics, which adds to the overall system complexity. Biocatalytic processes typically suffer from relatively slow reaction rates and require strictly controlled operating conditions, including optimal temperature and pH ranges, to maintain enzyme activity and stability. To address these challenges, coupled catalytic strategies that leverage the synergistic effects of multiple energy forms have emerged as a promising approach to achieve high efficiency, low energy consumption and enhanced product selectivity. Currently, multifield coupled systems, including photoelectro, photothermal, thermo‐electro, and biological‐electrochemical catalysis, are gaining significant attention in polyester wastes upcycling research. Meanwhile, these coupled systems also introduce new challenges, such as increased system complexity, higher costs, and greater difficulty in mechanistic understanding, all of which require further in‐depth investigation.
5. Incorporating TEA into the life cycle of polyester plastics within a circular economy framework is essential for identifying key cost drivers, potential benefits, risks, and opportunities. The sustainable development of a polyester circular economy fundamentally depends on economic viability. However, the transition of advanced technologies from laboratory research to large‐scale application often faces significant economic and market uncertainties. A systematic TEA approach can effectively reduce these uncertainties, facilitate the identification of technologies with genuine industrial potential, accelerate their commercialization, and promote the alignment of environmental sustainability with economic profitability.

Achieving both carbon neutrality and a circular polyester plastics economy will ultimately require the development of green synthesis, efficient recycling and high‐value utilization technologies for polyester plastics. Future progress depends on the integration of interdisciplinary innovations and full‐chain design strategies to realize a truly circular economy for polyester plastics.

## Conflict of Interest

The authors declare no conflict of interest.

## Author Contributions

Conceptualization, J.L. and J.Y.; writing original draft, J.L.; writing review and editing, J.C., Y.Z., P.G., and L.W., data interpretation and review, J.C., P.G., L.W., and J.Y.; copyright, J.L.; funding acquisition, J.Y.
